# Stem Cells and Bone Tissue Engineering

**DOI:** 10.3390/life14030287

**Published:** 2024-02-21

**Authors:** Xueqin Gao, Joseph J. Ruzbarsky, Jonathan E. Layne, Xiang Xiao, Johnny Huard

**Affiliations:** 1 Linda and Mitch Hart Center for Regenerative and Personalized Medicine, Steadman Philippon Research Institute, Vail, CO 81657, USA; jruzbarsky@thesteadmanclinic.com (J.J.R.); jlayne@sprivail.org (J.E.L.); 2 The Steadman Clinic, Aspen, CO 81611, USA; 3 Glassell School of Art, The Museum of Fine Arts, Houston, TX 77006, USA; xiangxiao6363@gmail.com

**Keywords:** bone tissue engineering, bone marrow mesenchymal stem cells, muscle-derived stem cells, adipose-derived stem cells, dental pulp stem cells, periodontal ligament stem cells, periosteum stem cells, umbilical cord-derived stem cells, peripheral blood stem cells, urine-derived stem cells, exosome, extracellular vesicles, microRNA, bone morphogenetic proteins

## Abstract

Segmental bone defects that are caused by trauma, infection, tumor resection, or osteoporotic fractures present significant surgical treatment challenges. Host bone autograft is considered the gold standard for restoring function but comes with the cost of harvest site comorbidity. Allograft bone is a secondary option but has its own limitations in the incorporation with the host bone as well as its cost. Therefore, developing new bone tissue engineering strategies to treat bone defects is critically needed. In the past three decades, the use of stem cells that are delivered with different scaffolds or growth factors for bone tissue engineering has made tremendous progress. Many varieties of stem cells have been isolated from different tissues for use in bone tissue engineering. This review summarizes the progress in using different postnatal stem cells, including bone marrow mesenchymal stem cells, muscle-derived stem cells, adipose-derived stem cells, dental pulp stem cells/periodontal ligament stem cells, periosteum stem cells, umbilical cord-derived stem cells, peripheral blood stem cells, urine-derived stem cells, stem cells from apical papilla, and induced pluripotent stem cells, for bone tissue engineering and repair. This review also summarizes the progress using exosomes or extracellular vesicles that are delivered with various scaffolds for bone repair. The advantages and disadvantages of each type of stem cell are also discussed and explained in detail. It is hoped that in the future, these preclinical results will translate into new regenerative therapies for bone defect repair.

## 1. Introduction

The repair of large bony defects that are caused by trauma, infection, tumor resection, or fractures has traditionally relied on the use of bone autograft or allograft. Technological advances have allowed for the development of alternative approaches for the repair of bone defects and fracture non-unions using tissue engineering strategies in combination with stem/progenitor cells, bone growth factors, and scaffolds of varying biomaterials. Many different types of stem cells, derived from various tissue sources, have been explored to promote stem cell-mediated bone regeneration with varying degrees of success. 

Adult or postnatal stem cells can be isolated from almost any tissue. The most commonly studied postnatal stem cells include bone marrow mesenchymal stem/stromal cells (BMMSCs), muscle-derived stem cells (MDSCs), adipose-derived stem cells (ADSCs), umbilical cord-derived mesenchymal stem cells (UC-MSCs), periosteal stem cells (PSCs), dental pulp-derived stem cells (DPSCs), periodontal ligament stem cells (PDLSCs), peripheral blood-derived mesenchymal stem cells (PB-MSCs), urine-derived system cells (UDSCs), stem cells from apical papilla (SCAP), and induced pluripotent stem cells (iPSCs). Most of the review articles only cover certain types of stem cells for bone tissue engineering; this review focuses on the most recent progress that has been made utilizing the above-mentioned stem cells for bone tissue engineering and regeneration. This review also discusses the advantages and disadvantages of different kinds of stem cells. Given the vast amount of available research, in vivo preclinical studies involving the combination of different stem cells and scaffolds for bone tissue engineering, with or without varying growth factor modifications, are the focus of this detailed review.

## 2. Bone Marrow Mesenchymal Stem (Stromal) Cells (BMMSCs)

Bone marrow cells are both the earliest and most frequently investigated stem cells for use in bone tissue engineering. In 1991, Connolly et al., first explored the use of autologous bone marrow aspirate (BMA) for the treatment of fracture non-unions. They found that the BMA stimulated callus formation and eventual union in 8/10 patients who underwent cast fixation and 10/10 patients who underwent intramedullary nail fixation. The authors concluded that bone marrow injection was as effective as open autologous bone grafting but with considerably fewer disadvantages [1]. Furthermore, combining BMMSCs with demineralized bone matrix has been shown to be as effective as autologous iliac crest bone grafting for the treatment of non-unions [2]. In addition, loading BMMSCs onto a hydroxyapatite scaffold has also been shown to promote new bone formation within bone defects, which may allow for the eventual recovery of skeletal function [2,3]. Over the past two decades, many in vitro and in vivo preclinical studies using animal models have demonstrated the effectiveness of using BMMSCs for bone tissue engineering. Combining BMMSCs with different scaffolds has been shown to be more efficient than applying a scaffold alone [4]. For the treatment of craniofacial defects, combining human BMMSCs with bone morphogenetic protein (BMP) 7 (an osteoinductive growth factor) improved the healing of the craniofacial bone defect [5]. In addition, Scotti C et al., proposed a novel strategy to enhance new bone formation by subcutaneously implanting a human BMMSC-derived cartilage intermediate. The group demonstrated that this cartilage intermediate formed a functional bone organ with an outer layer, mainly consisting of host cells which overlaid a premineralized area, and an inner, trabecular-like, endochondral bone layer. The regenerated bone contained bone marrow with sinusoid-like structures of blood vessels, multiple lineages of hematopoietic stem cells, and progenitor cells that were similar to that of native bone, which represents a strategy of a “developmental engineering” paradigm for functional bone regeneration [6,7]. Long T et al., showed that BMMSC cell sheets closely mimic the periosteum and allow osteogenic cells to promote new bone formation in critical-size bone defects as efficiently as autograft bone does [8]. 

Lin H et al., have already reviewed the progress of BMMSC aging and tissue engineering applications to enhance bone repair [9]. The clinical application of BMMSCs for skeletal tissues repair was also reviewed by Arthur A et al., and Stamnitz S et al. [10,11]. Therefore, this section of the review will mainly focus on progress made in the past 5 years of using human bone marrow stem cells for bone tissue engineering. 

### 2.1. BMMSCs Loaded with Different Scaffolds for Bone Tissue Engineering

Juan Francisco Blanco et al., reported that transplantation of human BMMSCs with a tri-calcium phosphate (TCP) scaffold into a rabbit femoral condyle critical-size bone defect achieved improved bone regeneration and better osteointegration, without eliciting inflammation, compared with the TCP scaffold alone [12]. Yang C et al., reported that rat BMMSCs transduced with lentiviral miRNA-21 and loaded within a TCP scaffold enhanced bone regeneration in a critical-size calvarial bone defect model in rats compared to the scaffold alone or the BMMSCs alone. Canine BMMSCs transduced with lentiviral miRNA-21 were also found to enhance alveolar bone regeneration. The enhanced osteogenesis and bone regeneration was attributed to the upregulation of P-AKT/HIF1α and the endogenous BMP2 expression [13]. Human BMMSCs seeded with gadolinium-doped bioglass (Gd-BG) scaffolds and fabricated by combining hollow mesoporous Gd-BG microspheres with chitosan demonstrated enhanced osteogenesis in vitro and enhanced critical-size defect healing in a rat cranial bone defect model [14]. Du FF et al., compared fresh, autologous bone marrow mononuclear cell concentrate (BMMNC) with BMMSCs for repairing a segmental femur bone defect (1.5 cm in length) in beagle dogs using β-TCP as a scaffold. The results showed that the BMMNC group promoted greater bone regeneration than the cultured BMMSC group. The grafts in the BMMNC group also demonstrated improved mineralization, with a collagen arrangement and micro-biomechanical properties that were more similar to the contralateral native tibia bone [15]. 

Human BMMSCs loaded into calcium-deficient hydroxyapatite (CDHA) scaffolds showed complete bone healing of a calvarial bone defect in nude mice with demonstrated bone formation in the pores of the CDHA scaffold. In contrast, when delivered with sintered β-TCP, the new bone formed along the periphery of β-TCP. CDHA scaffolds can be prepared at an ambient temperature, yielding closer-to-native bone mineralization than β-TCP scaffolding. Also, BMMSCs showed better engraftment when loaded onto CDHA scaffolding compared with the β-TCP [16]. Others reported that autologous BMMSCs and platelet-rich plasma (PRP) combined with a calcium phosphate cement (CPC) scaffold for femoral, critical-size bone defects resulted in healing in minipigs. The newly formed bone area was higher in the group with CPC scaffold-loaded MSCs and PRP than in the CPC scaffold-only group at each time point (all *p* < 0.05). Thus, the strategy of CPC combined with BMMSCs and PRP may also be an effective method to repair bone defects [17]. 

Lin H et al., reported using a new nanocomposite that incorporated the graphene oxide (GO)-based nanosheets or silica-coated GO (SiGO) into methacrylated gelatin (GelMA)-based scaffolds to evaluate bone formation by human BMMSCs. The incorporation of GO markedly increased mineralization within human BMMSC-laden constructs, which was further increased by replacing GO with SiGO. A mechanistic analysis revealed that the nanosheet enhanced the production, retention, and biological activity of endogenous BMPs, resulting in robust osteogenesis in the absence of exogenous osteoinductive growth factors. The bone formation potential of this technology was further tested in vivo using subcutaneous implantation in a mouse model, which revealed that human BMMSC-laden GO/GelMA and SiGO/GelMA samples resulted in bone volumes that were 108 and 385 times larger, respectively, than the GelMA control group. These results demonstrated the biological activity and mechanism of action of GO-based nanosheets in augmenting the osteogenic capability of human BMMSCs and highlighted the potential of leveraging nanomaterials such as GO and SiGO for bone tissue engineering applications [18]. The same group also constructed a pre-vascularized bone-like tissue using human BMMSCs embedded in their own self-generated extracellular matrix. Then, a 3D culture of human umbilical vein endothelial cells (HUVECs)/HBMSCs was introduced to cover a bone-like construct’s surface for vascularization. The authors showed that this endochondral ossification-inspired procedure resulted in a robust osteogenic differentiation of human BMMSCs and markedly promoted the HUVEC/HBMSC network’s formation in vitro. When the pre-vascularized bone-like tissues were subcutaneously implanted into mice, they exhibited significantly more functional blood vessel formation than the control group that contained a single type of HUVEC and HBMSC cells, along with increased bone formation and remodeling [19]. Others demonstrated that GO-modified natural biocompatible protein Bombyx mori silk fibroin (SF) accelerated the early cell adhesion and induced the osteogenic differentiation of human BMMSCs even in the absence of additional osteogenic inducers in the medium [20].

Several recent studies also highlight the potential for specialized scaffold engineering using a variety of materials and production methods. BMMSCs differentiated on silk scaffolds into either hypertrophic chondrogenic pellets or osteogenic pellets were shown to form immature and mature bone when implanted subcutaneously, although the hypertrophic chondrogenic pellets formed more vascularized bone compared with osteogenic pellets, thus better mimicking the endochondral ossification process [21]. Rat MSCs loaded with a photo-crosslinked biomimetic GelMA hydrogel scaffold resulted in robust new bone formation in a rat femoral bone defect model [22]. Machado-Paula et al., combined polycaprolactone (PCL) fibers, carbon nanotubes (CNTs), and hydroxyapatite nanoparticles (nHap) and demonstrated that the fibers that were formed by rotary jet spinning (RJS) instead of traditional electrospinning (ES) with embedded rat BMMSCs showed the best outcomes in terms of repairing rat calvarial bone defects after 6 weeks, as demonstrated by a 10-fold increase in new bone formation compared to the RJS scaffold or BMMSCs only in a rat 5 mm critical-size bone defect [23]. 

Liu X et al., also engineered scaffold-free, functional spheroids with rat MSCs and two-dimensional hetero-nano-layers (2DHNLs), consisting of black phosphorus (BP) and GO, to create a 3D cell-instructive microenvironment for large bone defect repair. After transplantation of the spheroids into the critical-size calvarial defects of rats, the authors demonstrated enhanced bone regeneration and neo-vascularization, as well as improved support for the osteogenic differentiation of the rat MSCs. Furthermore, adding the osteogenic factor, dexamethasone (DEX), on the 2DHNL showed high in vivo osteogenic induction and bone regrowth without prior in vitro culture in osteogenic medium. These functional, 2DHNL-impregnated spheroids that enable rat MSCs and osteogenic factor co-delivery could be a promising strategy for effective in vivo bone repair [24]. Hang Lin’s group developed an innovative idea by seeding human BMMSCs on their own secreted extracellular matrix (mECM) without an exogenous scaffold to allow human BMMSCs to undergo the N-cadherin-mediated developmental condensation process and subsequent chondrogenesis. Furthermore, the BMMSC-mECM constructs significantly enhanced bone formation in vivo via endochondral ossification [25]. It has also been shown that locally delivered, fluorescent nanoparticle (fNP)-labeled murine MSCs enhanced tibial defect repair, increased the number of stem cells, and supported vascular maturation in mice. fNP-MSCs also survived in the defect throughout repair. While only a small portion of the transplanted cells underwent osteogenic differentiation (OSX+), a significant portion maintained their expression of MSC and skeletal stem cell markers (SCA-1 and PRRX1) [26]. Pitacco P et al., designed a new bone tissue engineering strategy of incorporating human BMMSCs into fibrin-based bioinks and then bioprinting these into PCL frameworks to produce mechanically reinforced constructs. Different in vitro culture regimens were used to produce chondrogenic and early hypertrophic engineered grafts. Following their implantation into femoral bone defects of transiently immunosuppressed rats, the bioprinted constructs rapidly remodeled into bone in vivo, with early hypertrophic constructs supporting higher levels of vascularization and bone formation compared to the non-hypertrophic chondrogenic constructs. The early hypertrophic bioprinted constructs also supported higher levels of vascularization and spatially distinct patterns of new bone formation compared to BMP2-loaded collagen scaffolds (positive control) [27]. Shuai Y et al., designed an artificial periosteum that nucleates and generates hydroxyapatite crystals on the surface of the Antheraea pernyi fibroin (AF) membrane to generate a mineralized AF membrane (MAF). This artificial periosteum (MAF) significantly promoted osteogenic differentiation of human MSCs in the absence of an osteogenic inducer in vitro. In vivo results demonstrated that bone-related matrix proteins are highly expressed in MAF groups with or without seeded MSCs [28]. Zhang Q et al., developed an air plasma-treated natural silk fibroin (SF) membrane to enhance the osteogenic differentiation of rat BMMSCs. The air plasma-treated SF films (termed A-SF) exhibited surface nano-pillars and enhanced hydrophilicity compared to the pristine SF films (termed SF), making the A-SF and SF films induce the formation of plate-shaped/more crystalline and needle-like/less crystalline nHAp, respectively. The A-SF-nHAp and A-SF films exhibited more efficient bone formation than the SF-nHAp and SF films at 4 weeks due to their unique nanotopography, with the A-SF-nHAp films being more efficient than the A-SF films [29].

### 2.2. BMMSCs Delivered with Scaffold and Bone Growth Factors for Bone Tissue Engineering

Herberg S et al., designed MSC tube condensates to mimic the femoral diaphysis, then applied transforming growth factor (TGF)-β1 and BMP2, attempting to recapitulate endochondral ossification. First, localized TGF-β1 + BMP2-morphogen delivery stimulated chondrogenic priming/endochondral differentiation of the tubular condensates in vitro. When implanted subcutaneously, the human BMMSC tubes formed cartilage templates that underwent bony remodeling. Application of the MSC tubes was found to stimulate more robust endochondral defect healing than human BMMSC sheets, without significant ectopic bone formation, in a segmental femoral defect model [30]. BMP-6-loaded nano-hydroxyapatite (nHA)/gelatin (Gel)/gelatin microsphere (GMS) scaffolding, pre-seeded with BMMSCs, was shown to promote BMMSC osteogenic differentiation in vitro and significantly accelerated bone regeneration in a rat critical-size calvarial defect model [31]. 

Human BMMSC spheroids loaded with BMP2-HA in viscoelastic gels demonstrated greater calcium deposition than human BMMSC spheroids entrapped in elastic alginate gels. After implantation into critical-size calvarial bone defects, the viscoelastic hydrogels encased with human BMMSC spheroids appeared to be a more potent stimulator of osteogenesis than hydrogels with BMP2-HA alone. However, increases in bone formation were evident in the viscoelastic gels, regardless of the BMP2 integration method (i.e., soluble delivery versus HA nanoparticles) [32]. Yang M’s group designed chimeric peptide with one peptide motif that was bound to Bombyx mori silk fibroin (SF) membrane (SFm) and fused to peptide with the goal to promote osteogenesis in vitro and bone formation in vivo. The chimeric peptide enabled SFm to effectively induce osteogenic differentiation of human BMMSCs, even without other osteogenic inducers, and efficiently stimulated bone regeneration in a subcutaneous rat model in 8 weeks, even without MSC seeding, and not eliciting inflammatory responses [33].

### 2.3. BMMSC-Derived Exosomes or Extracellular Vesicles in Bone Tissue Engineering

Fan J et al., developed an approach to amass exosome mimetics (EMs) from human BMMSCs. The human BMMSCs-EMs had a significantly increased proportion of vesicles that were positive for the exosome-specific CD63 marker when compared with BMMSC-derived exosomes using the traditional method and demonstrated enhanced bone regeneration in vivo using a chitosan hydrogel scaffold. Further, EMs from noggin knockdown BMMSCs enhanced bone regeneration via the inhibition of miR-29a [34]. Another study showed that human BMMSCs that were osteogenically pre-differentiated for 10 and 15 days led to the production of osteogenic exosomes. When the purified exosomes were loaded into the 3D-printed titanium alloy scaffolds, the cell-free, exosome-coated scaffolds regenerated bone tissue as efficiently as human BMMSC-seeded exosome-free scaffolds within 12 weeks. Furthermore, RNA sequencing indicated that the osteogenic exosomes induced the osteogenic differentiation by using their cargo, including upregulated osteogenic miRNAs (Hsa-miR-146a-5p, Hsa-miR-503-5p, Hsa-miR-483-3p, and Hsa-miR-129-5p) or downregulated anti-osteogenic miRNAs (Hsa-miR-32-5p, Hsa-miR-133a-3p, and Hsa-miR-204-5p), and by activating the PI3K/Akt and MAPK signaling pathways [35]. The rat BMMSC-derived exosome (BMSC-OI-exosome) contains the miRNAs let-7a-5p, let-7c-5p, miR-328a-5p, and miR-31a-5p, which target Acvr2b/Acvr1 and regulate the competitive balance of Bmpr2/Acvr2b toward Bmpr-elicited Smad1/5/9 phosphorylation. The delivery of the BMSC-OI-exosome using mesoporous bioactive glass (MBG) rapidly induced bone regeneration in a rat skull defect model [36]. In a different study, BMMSC-derived functionally engineered EVs (FEEs) could bind to mimetic peptides from collagen (DGEA, GFPGER) and fibronectin (RGD). Using photo-crosslinkable alginate hydrogels containing RGD to encapsulate, tether, and retain the FEEs over a period of 7 days helped maintain the structural integrity and osteoinductive functionality of the EVs. When implanted in a calvarial defect model in vivo, alginate–RGD hydrogels containing the FEEs enhanced bone regeneration 4-fold when compared to controls lacking FEEs, and 2-fold when compared to controls without the tethering peptide [37]. 

### 2.4. Targeting Cell Senescence to Improve BMMSC-Mediated Bone Tissue Engineering

Cell senescence due to serial in vitro expansion can negatively affect MSCs’ functionality when implanted in vivo for bone defect healing. Therefore, eliminating the senescent cell burden before cell transplantation represents a new strategy to enhance BMMSC-mediated bone defect healing. For example, Xu M’s group reported that treatment of aged murine BMMSCs with dasatinib plus quercetin improved bone regeneration of aged murine BMMSCs [38]. Xing X et al., screened senolytic drugs using induced–senescent BMMSCs and found that quercetin, among others, can reliably eliminate senescent MSCs during culture, restore the MSC self-renewal potential, and promote osteogenic differentiation in vitro. In vivo, quercetin, when delivered with a hydrogel locally, enhanced long bone and calvarial defect healing in aged rats by decreasing MMP secretion from the local environment [39]. 

Taken together, BMMSCs combined with different scaffolds and osteogenic factors are the most commonly used for bone tissue engineering and are summarized in Supplemental Table S1. 

## 3. Muscle-Derived Stem Cells (MDSCs)

David Yaffe first isolated myoblasts from rat skeletal muscle tissues using pre-plate techniques [40]. These cells could be cultured in vitro for many months, maintaining their capacity for continuous proliferation, fusion, and differentiation into postnatal multinucleated myofibers. Virtually all the cells in this cell line have the potential to differentiate into myofibers [40]. Later, Dr. Yaffe established a C2 myoblast cell line from the injured thigh muscle of two-month-old C3H mice [41]. Blau H et al., further re-cloned C2 cells and expanded the cells to the C2C12 myoblast cell line [42]. Furthermore, Blau’s group isolated primary myoblasts using the pre-plating method and found that these cells are primarily myogenic, both in vitro and in vivo [43]. Subsequently, the Huard group isolated a subpopulation of muscle stem cells, termed muscle-derived stem cells (MDSCs, pre-plate 6), using the modified pre-plate technique, demonstrating that MDSCs are not only myogenic, but also multipotent in vitro and in vivo [44,45,46,47]. 

An earlier study from the Huard group demonstrated that after transduction with BMP2 using an adenoviral vector, the murine MDSCs were found to be very efficient at regenerating new bone within a critical-size calvarial bone defect created in mice [45]. Additionally, it has been demonstrated in vivo that transplantation of allogenic retroviral BMP-4-transduced murine MDSCs regenerated bone efficiently in both immunocompetent mice, using a heterotopic bone formation model, and in a critical-size calvarial bone defect model in immunocompromised mice [48]. Further studies found that the transplantation of murine MDSCs with retrovirally transduced BMP4 or BMP2 in combination with retrovirally transduced VEGFα further enhanced retro-BMP2-mediated bone regeneration by promoting angiogenesis. Vice versa, when retrovirally BMP2-transduced murine MDSCs were co-transplanted with retroviral soluble fms-like tyrosine kinase 1 (sFlt1), a soluble receptor of VEGFA, bone regeneration was inhibited [49,50]. The bone regeneration capacity of murine MDSCs can also be fine-tuned by co-transplanting murine MDSCs that are transduced to express BMP4 and noggin (BMP antagonist), with the latter preventing bone overgrowth [51]. In addition, retro-BMP4/green fluorescent protein (GFP) murine MDSCs not only healed critical-size bone defects via their direct differentiation into osteoblasts and osteocytes, they also influenced the host cells’ responses through the secretion of various paracrine factors (MCP1, VEGFα, FGF2, IGF2, PDGF, and TGFβ1) [52]. The capacity of murine MDSCs to promote new bone formation is also affected by the sex of the donor and recipient. Male MDSCs were found to be more efficient than female MDSCs at promoting osteogenic differentiation in vitro and new bone formation in vivo [53]. Notably, this gender difference in murine MDSCs in bone formation was not related to variations in the concentration of circulating hormonal factors between male and female recipients [54]. 

Human MDSCs isolated using the pre-plate technique and transduced with BMP2 using retroviral and adenoviral vectors were able to promote the healing of critical-size calvarial defects in SCID mice, as demonstrated by gross observation and histology; additionally, a small fraction of the cells were found to contribute to bone formation [55]. Mastrogiacomo M et al., isolated human skeletal muscle-derived cells (MDCs) using a similar technique and found that the human MDCs underwent osteogenic, chondrogenic, and adipogenic differentiation in vitro and formed new bone and cartilage in vivo when implanted subcutaneously [56]. Gao X et al., reported successful isolation of hMDSCs using pre-plate techniques and found that 95% of hMDSCs express CD73, CD90, CD105, and CD44. Furthermore, 95% of the hMDSCs are also positive for CD56 and CD146, while also being negative for UEA and CD45 [57]. These cells did not display a small, round, and shiny morphology as reported for mMDSCs, but rather, they showed a spindle-like, MSC-like morphology [57]. The hMDSCs also underwent adipogenesis, chondrogenesis, osteogenesis, and myogenesis [57]. When the hMDSCs were transduced with lentiviral BMP2, they regenerated a substantial amount of new bone and healed more than 75% of critical-size bone defects in immunocompromised mice [57]. It was further demonstrated that hMDSCs transduced with lentiviral BMP2 were just as efficient as human BMMSCs in terms of regenerating new, functional bone in a critical-size calvarial bone defect model [58]. Interestingly, bone regeneration with lentivirally BMP2-transduced hMDSCs is not affected by the donor cells’ age but is impaired by the host’s age, which indicated that the host micromilieu is also important for stem cell-mediated bone regeneration [59]. Also, the quality of the bone that is formed from retro-BMP4 transduced mMDSCs is dependent on the scaffold that is used for delivery. Usas A et al., compared Gelfoam, a collagen gel (CG), and fibrin sealant (FS) as scaffolds and found that FS- and CG-based scaffolds healed calvarial bone defects with a closer resemblance to native bone compared with the bone overgrowth that was observed in the Gelfoam group. The FS scaffolds induced less ectopic ossification, further demonstrating fine-tuned MDSC delivery via absorbable bioengineered scaffold delivery systems. However, none of these scaffolds induced new bone formation when loaded with mMDSCs that were transduced with LacZ (reported gene), which indicated the importance of BMP in inducing mMDSC-mediated bone formation [60]. 

Human muscle-derived cells isolated using cell sorting techniques have also shown bone regeneration capacity. For example, Zheng B et al., found a population of myogenic endothelial stem cells (sorted for CD56^+^CD34^+^CD144^+^CD45^−^) that could promote new bone formation in a muscle heterotopic bone formation model when transduced with BMP2 [61,62]. Another group has shown that the MSCs that can be isolated from traumatically injured human muscles were also able to differentiate into osteoblasts, adipocytes, and chondrocytes [63]. 

Because orthopedic trauma often involves the disruption of both muscle and bone within localized areas, it is possible that skeletal muscle tissue could be an important source of MSCs for future applications in orthopedic trauma surgery (see Supplemental Table S1). 

## 4. Adipose-Derived Stem Cells (ADSCs)

The use of adipose-derived stem cells (ADSCs) has garnered significant research interest in the field of tissue engineering, because they are easily harvested and accessible in human patients. Since the techniques that are used for the isolation, characterization, and cryopreservation of ADSCs for applications in other areas of tissue engineering have been thoroughly reviewed by Levi B, Longaker MT [64], and De Francesco F [65], this section will only focus on the use of ADSCs for bone regeneration.

### 4.1. ADSCs Alone with Scaffold for Bone Tissue Engineering

The first in vivo study that investigated the bone-regenerative capacity of murine ADSCs seeded on apatite-coated PLGA scaffolds was performed by Longaker’s group [66]. The authors found that, in both juvenile and adult mice, transplantation of non-transduced ADSCs produced large, yet comparable, amounts of new bone in calvarial bone defects when compared to bone marrow cells and osteoblasts [66]. It was also shown that the transplanted ADSCs contributed to 84–99% of the new bone formation in the defective area [66]. In the same year, a case report demonstrated that autologous fibrin glue loaded with ADSCs significantly promoted the healing of a traumatic calvarial defect [67]. Furthermore, it has been shown that the capacity of ADSCs to induce new bone formation is compromised by the freezing and subsequent thawing of the cells [68]. In vitro studies revealed a significant negative impact of the freeze–thaw process on cell proliferation, as well as osteogenic and adipogenic differentiation (*p* < 0.01) [68]. In vivo experiments showed near-complete healing in calvarial defects treated with fresh human adipose stem cells (hADSCs) in contrast to the minimal healing observed with freeze–thaw hADSCs (*p* < 0.01) [68]. However, adding either recombinant insulin-like growth factor 1 (rIGF1) or recombinant BMP4 (rBMP4) significantly offset the impaired osteogenic differentiation in frozen hADSCs (*p* < 0.01) [68]. Kim Y et al., investigated the bone regeneration potential of canine ADSCs and an ADSC osteogenic cell sheet (OCS) on a critical-size radial segmental defect (15 mm long) in a canine model using composite PCL/β-tricalcium phosphate (β-TCP) scaffolds. The study revealed that the combination of an OCS with aPCL/β-TCP composite scaffold maximized the new bone mass volume (28.11 ± 5.5 cm^3^) in canine radial defects, outperforming PCL/β-TCP with undifferentiated ADSCs, with osteogenic ADSCs, and alone. Though the defect was not completely healed in any of these groups, this study highlights the superior efficacy of an OCS in conjunction with a composite scaffold for enhanced bone regeneration in critical-size defects [69]. 

Orbay H et al., conducted a study using ADSCs harvested from the inguinal fat pads of male Lewis rats, differentiating them toward endothelial and osteoblastic lineages before transplantation into critical-size calvarial defects. The rats (n = 30) were randomized into four groups, utilizing hydroxyapatite/poly(lactide-co-glycolide) [HA-PLG] scaffolds alone or scaffolds seeded with non-differentiated ADSCs, ADSC-derived endothelial cells, or ADSC-derived osteoblasts. Micro-CT analysis 8 weeks post-operation revealed the highest bone mineral density in the ADSC-derived osteoblast group (1.46 ± 0.01 g/cm^3^), followed by the ADSC-derived endothelial cell group (1.43 ± 0.05 g/cm^3^), the scaffold-only group (1.42 ± 0.05 g/cm^3^), and the non-differentiated ADSC group (1.3 ± 0.1 g/cm^3^) [70]. Although the osteogenically differentiated ADSC group exhibited the highest vascular density, the differences among the groups did not achieve statistical significance (*p* > 0.05), indicating that ADSC-derived endothelial cells and osteoblasts provided a limited increase in calvarial bone healing when combined with HA-PLG scaffolds [70]. Bernhard J et al., created tissue-engineered grafts using human ADSCs by differentiating them into hypertrophic chondrocytes within decellularized bone scaffolds and compared these to acellular scaffolds and grafts engineered using ADSC-derived osteoblasts. After implanting these grafts into critical-size femoral defects in athymic rats for 12 weeks, the grafts that were engineered using hypertrophic chondrocytes recapitulated endochondral ossification [71]. Highly enhanced bone deposition was associated with extensive bone remodeling and the formation of bone marrow, as well as with the presence of pro-regenerative M2 macrophages within the hypertrophic grafts [71]. As a result, hypertrophic chondrocyte grafts bridged seven-eighths of defects, compared to only one-eighth for osteoblast grafts and three-eighths for acellular scaffolds. These results suggested that the ADSC-derived hypertrophic chondrocytes in osteogenic scaffolds can markedly improve long bone repair [71]. Liu J et al., investigated rat allogeneic ADSCs combined with heterogeneous deproteinized bone (HDB) to repair segmental 4 mm radial defects in rats. The authors found that ADSCs-HDB with an in vitro pre-osteogenic differentiation group regenerated the radial defects completely in 8 weeks. The ADSCs-HDB group without pre-differentiation also promoted bone defect healing compared to the HDB scaffold and blank control groups, with the blank control group resulting in a non-union. These results indicate that in vitro pre-osteogenic differentiation of ADSCs is more effective for promoting bone defect healing than using undifferentiated ADSCs, and it represents an effective way for bone tissue engineering [72]. In another study, hADSCs were seeded in 3D culture systems, using spheroids and polystyrene scaffolds to mimic the native stem cell niche. The spheroids, in particular, exhibited enhanced osteogenic differentiation, as evidenced by the ALP activity and the upregulated expression of key osteogenic markers such as RUNX2, osterix, integrin-binding sialoprotein (IBSP), and osteocalcin compared to both the polystyrene scaffolds and traditional 2D culture [73].

Zhang H et al., used a rabbit ADSC double cell sheet (DCS) and a composite scaffold made with polylysine (PLL)-modified coralline hydroxyapatite (CHA) with the aim of engineering vascularized bone to repair large-radius bone defects in rabbits. At 12 weeks after surgery, the defective surface of the DCS-PLL-CHA group was completely wrapped by bone tissue and osteoids, the cortical bone was continuous, and the medullary cavity was perforated. A large amount of well-organized lamellar bone was formed, a small amount of undegraded CHA exhibited a linear pattern, and a significant amount of bone filling could be seen in the pores. Furthermore, although the expression levels of BGLAP, SPP1, and VEGF were similar in each group, the PECAM1 expression was higher in the DCS-PLL-CHA group than in the autogenous bone group and the CHA group [74]. Pig ADSCs seeded with tricalcium phosphates (TCPs) and a PLGA scaffold enhanced mandibular bone defect healing in miniature pigs, exhibiting a significantly higher bone volume percentage being regenerated (34.8% ± 4.80%) than scaffolds implanted without cells (n = 6, 22.4% ± 9.85%), as revealed by micro-CT (*p* < 0.05). Moreover, an increased amount of osteocalcin deposition was found in the experimental group in comparison to the control group (27.98 ± 2.81% vs. 17.10 ± 3.57%, *p* < 0.001) [75]. 

### 4.2. ADSCs Modified with Different Growth Factors for Bone Tissue Engineering

Peterson B et al., found that human processed lipoaspirate (HPLA) cells that were genetically modified to overexpress BMP2 induced the complete healing of femoral defects within a period of 8 weeks [76]. In contrast, the implantation of HPLA cells that were not transduced to overexpress BMP2 resulted in no significant new bone formation. BMP2 alone also healed the femoral defect [76]. Hsu MN et al., designed a hybrid baculovirus (BV) system for the delivery of the BMP2 gene and the CRISPRi system targeting the noggin of rat ADSCs. After BV-mediated co-delivery into ADSCs, the system induced prolonged BMP2 expression and simultaneously stimulated Nog expression, while the CRISPRi system effectively repressed Nog upregulation for at least 14 days. The CRISPRi-mediated Nog knockdown, along with BMP2 overexpression, further stimulated the osteogenic differentiation of ADSCs. The implantation of the CRISPRi-engineered ADSCs into critical-size defects at the calvaria significantly enhanced the calvarial bone healing and matrix mineralization [77]. Chou YF et al., incorporated different doses of recombinant human BMP2 (rhBMP2) onto apatite-coated porous poly(l-lactide-co-dl-lactide) (70:30) (PLDLA) scaffolds, seeded them with ADSCs, and then implanted them into athymic rats to observe the critical-size femoral defect healing. Interestingly, the results showed that the combination of ADSCs and rhBMP2 onto a scaffold did not enhance the healing of calvarial defects when compared to rhBMP2 alone. This result may indicate that at certain dosages, rhBMP2 plays a dominant role in bone regeneration, potentially masking the therapeutic effects of implanted stem cells [78]. 

Kim Y. et al., used lentivirus-mediated BMP7-transduced ADSCs to osteogenically enhance cell sheets that were loaded with a PCL/β-TCP scaffold or combined with a demineralized bone matrix (DBM). When applied in a 15 mm long, segmental radius defect in a canine model, the BMP7-overexpressing cell sheets, particularly when used with a DBM, significantly increased bone regeneration and vascularization, evidenced by micro-CT analysis, histological evaluation, and gene expression analyses [79]. Osinga R et al., developed a strategy to use hADSCs to regenerate bone through endochondral ossifications by inducing chondrogenesis in vitro before transplantation. ADSCs were cultured either as micromass pellets or into collagen sponges in a chondrogenic medium containing TGF-β3 and BMP6 for 4 weeks (early hypertrophic templates) or for 2 additional weeks in a medium supplemented with β-glycerophosphate, l-thyroxin, and interleukin1-β to induce hypertrophic maturation in vitro [80]. When transplanted in vivo, both the early and late hypertrophic templates underwent cartilage remodeling, as assessed by MMP13 and tartrate-resistant acid phosphatase-positive staining, and developed bone ossicles, including bone marrow elements, although to variable degrees of efficiency. In situ hybridization for human-specific sequences and staining with a human-specific anti-CD146 antibody demonstrated the direct contribution of ADSCs to bone and stromal tissue formation through endochondral ossification [80]. Lee J et al., developed PDGF- and bio-mineral-coated fibers which were then assembled with hADSCs to form spheroids, aiming to enhance vascularization alongside osteogenesis. When transplanted in vivo into a mouse calvarial defect, the spheroids demonstrated an enhanced bone regeneration area (42.48 ± 10.84%) and the greatest number of capillaries and arterioles derived from transplanted hADSCs [81]. Wang Z et al., evaluated the effects of drilling through the growth plate and using ADSCs and BMP2 to treat femoral head epiphyseal ischemic necrosis in a rabbit model. The authors found that the combination of growth plate drilling with either ADSCs or both ADSCs and BMP2 significantly enhanced the restoration of normal hip structures, improving femoral epiphyseal quotients and trabecular areas compared to controls and drilling-only groups (*p* < 0.01) [82]. The drilling plus BMP2 group also demonstrated improved femoral epiphyseal quotients and trabecular areas compared with those of untreated and drilling treatment-only groups (*p* < 0.05) [82]. 

Other studies used small molecules to enhance ADSC-mediated bone tissue engineering. Fan J et al., used the small molecule phenamil alongside BMP2 to promote in vitro osteogenic regeneration and enhance calvarial defect regeneration in a mouse model. It was found that treatment of ADSCs with BMP2 in combination with phenamil significantly promoted the in vitro osteogenic differentiation of ADSCs. In vivo, the scaffolds that were treated with phenamil + BMP2 significantly promoted mouse calvarial regeneration compared with the groups that were treated with phenamil or BMP2 alone [83]. Moreover, the combination treatment reduced the BMP2 dose without compromising the calvarial healing efficacy [83]. Yao W et al., developed a bone-seeking molecular compound, LLP2A-Alendronate (LLP2A-Ale), to augment ADSCs homing to bone, with the goal of accelerating bone healing in a mouse closed-fracture model. Mice with mid-femur fractures were treated with placebo, LLP2A-Ale (500 μg/kg, IV), ADSCs derived from wild-type female Osx-mCherry adipose tissue (ADSC, 3 × 105, IV), or ADSC + LLP2A-Ale. LLP2A-Ale treatment increased the number of exogenous ADSCs homing to the fracture gaps, enhanced the incorporation of these cells into the bone callus, and stimulated endochondral bone formation [84]. Additionally, higher engraftment of exogenous ADSCs in fracture gaps seemed to contribute to the overall fracture healing and improved bone strength [84]. Another study focused on the modulation of the Wnt signaling pathway, targeting inhibitory factors such as Dickkopf-1 (DKK1), a secreted Wnt pathway antagonist. Utilizing anti-DKK1 neutralizing antibodies increased the osteogenic differentiation of hADSCs in vitro. In vivo, systemic anti-DKK1 treatment improved hADSC engraftment, survival, and vascular ingrowth when implanted into femoral segmental bone defects in NOD-SCID mice compared with the isotype antibody control during the repair process [85]. The modulation of the paracrine effects that are seen in ADSC-based therapies also plays a significant role in bone regeneration. For example, Levi B et al., conducted in vitro and in vivo experiments combining hADSCs with mouse calvarial osteoblasts in a co-culture that was supplemented with Hedgehog modifiers and with an osteoconductive scaffold into calvarial defects in mice. Micro-CT, histological evaluation, in situ hybridization, and PCR revealed that hADSCs can promote bone defect healing via paracrine effects and the activation of the Hedgehog signaling pathway [86]. Taken together, ADSCs combined with different growth factors or inhibiting negative bone regeneration factors and delivered with a scaffold further enhanced the bone-regenerative potential of ADSCs. 

### 4.3. ADSC-Derived Exosomes for Bone Tissue Engineering

Human ADSC-derived exosomes that are immobilized on the polydopamine-coating poly (lactic-co-glycolic acid) (PLGA/pDA) scaffolds under mild chemical conditions can be slowly and consistently released in vitro and promote the osteogenic differentiation, proliferation, and migration of human MSCs [87]. In vivo results showed that this cell-free system significantly enhanced bone regeneration in a critical-size bone defect model, contributing in part to its osteoinductive effects, as well as its promotion of MSC migration and homing to the newly formed bone tissue [87]. The enrichment of hADSC-derived exosomes with miR-375, achieved through stable overexpression after lentiviral transfection, has been shown to improve the osteogenic differentiation of human BMMSCs and promote bone regeneration in rat calvarial bone defects [88]. Another study combined human ADSC-derived exosomes with Mg2^+^ and gallic acid (GA) and constructed a functionalized, cell-free PLGA/Exo-Mg-GA metal–organic framework (MOF). The composite demonstrated enhanced osteogenic, angiogenic, and anti-inflammatory capabilities. In vivo experiments further corroborated the osteogenic effects of this unique composite, revealing promotion of new bone formation and satisfactory osseointegration by stabilizing the bone graft environment, increasing the blood supply, promoting the osteogenic differentiation of endogenous cells, and accelerating bone regeneration [89]. Additionally, small extracellular vesicles (sEVs) derived from ADSCs and functionalized with a bioactive pentapeptide (cysteine–arginine–glutamic acid–lysine–alanine) (CREKA), showed increased binding to fibrin–fibronectin in vitro [90]. The functionalized sEVs also demonstrated enhanced bone repair capabilities in a 2.8 mm femur epiphyseal bone defect in vivo. These positive effects on bone repair were mediated by the modulation of local inflammation and enhancement of angiogenesis, osteogenesis [90].

### 4.4. miRNA-Regulated ADSCs for Bone Tissue Engineering

Human ADSCs overexpressing miR-450b have been shown not only to promote osteogenic differentiation in vitro, but also to enhance ectopic bone formation in vivo. This was achieved through the downregulation of BMP3, an abundant BMP member that inhibits bone formation [91]. Wang F et al., investigated the role of miR-150-5p in ADSC-mediated bone regeneration. ADSCs were transfected with miR-150-5p inhibitors, miR-150-5p ADV, or short hairpin RNA (shRNA) of Notch3, and the subsequent effects on osteogenesis were evaluated. A combination of hydroxyapatite/tricalcium phosphate (HA/TCP) ceramic powders and transfected ADSCs was implanted into BALB/C nude mice to study the in vivo effects. It was revealed that compared to the negative control (NC) and miR-150-5p overexpression groups, the inhibition of miR-150-5p (miR-150-5p ADV group) significantly increased ADSC osteogenesis by regulating Notch3. MiR-150-5p overexpression decreased the expression of pFAK, pERK1/2, and RhoA. Conversely, these expression levels were upregulated when miR-150-5p was inhibited or Notch3 was silenced [92]. Furthermore, miR-150-5p inhibition partially reversed the suppressive effect of notch3 knockdown on osteogenesis in vitro and in vivo. This study demonstrated that the combination of ADSCs with miR-150-5p inhibition and a HA/TCP scaffold might be a promising strategy for bone defect repair [92]. 

In summary, ADSCs combined with different bioengineered scaffolds, growth factors, and their exosomes are promising for bone tissue engineering due to their rich abundance. Studies that were described in this section are summarized in Supplemental Table S1.

## 5. Dental Pulp Stem Cells and Periodontal Ligament Stem Cells

The study of human dental pulp-derived stem cells (hDPSCs) or periodontal ligament cells (PDLSCs) for tissue engineering has been a topic of increasing interest. Several reviews have been published that summarize the numerous characteristics of stem cells that are derived from human dental tissues and that possess multipotency in vitro and may have significant implications in the field of regenerative medicine [93,94,95,96]. This section will mainly review the progress over the past 5 years. 

### 5.1. Unmodified DPSCs Loaded with Different Scaffold for Bone Tissue Engineering

Laino G pioneered the isolation of stem cells from human dental tissues using a c-kit^+^/CD34^+^/CD45^−^ marker profile (via fluorescence-activated cell sorting [FACS]) and demonstrated their self-renewal and multipotent differentiation capabilities, which facilitated the formation of living autologous fibrous bone tissue (LAB) in vitro. After implantation into immunocompromised mice, LAB contributed to the formation of lamellar bone composed of osteocytes [97]. Various studies have subsequently explored the osteogenic differentiation of DPSCs. 

Zhang W et al., demonstrated that both rat and human DPSCs and BMMSCs can efficiently undergo osteogenic differentiation in vitro; however, only rat BMMSCs underwent in vivo bone formation when seeded on hydroxyapatite/TCP scaffolds [98]. Interestingly, another group found that the DPSCs underwent osteogenic differentiation much more efficiently than BMMSCs [99]. It has also been shown that un-transduced DPSCs seeded onto collagen gel constructs can improve the healing of calvarial bone defects in the absence of growth factor supplementation. These DPSCs generated new bone via direct differentiation into an osteogenic lineage and stimulation of angiogenesis from the host tissue [100]. Rabbit autologous DPSCs expressed vimentin and CD44 when used (1 × 10^8^) with Bio-Oss^®^ scaffolding in the alveolar bone defects of rabbit toothless jaws, regenerating bone with abundant osteoblasts compared to the few osteoblasts that were seen in the scaffold-only group [101]. Zhang W et al., combined human DPSCs with tyrosine-derived polycarbonate polymer scaffolds E1001(1K) containing beta-tricalcium phosphate (β-TCP) [E1001(1K)/β-TCP] and compared this group to BMP2 (4 µg) in a rat mandibular ramus critical-size bone defect repair model. Human DPSC-seeded acellular E1001(1K)/β-TCP scaffolds were cultured in vitro in osteogenic media for 1 week before implantation. Live micro-CT imaging at 3 and 6 weeks post-implantation revealed robust bone regeneration in the BMP implant group. DPSCs seeded with higher (5 × 10^5^) and lower density (2.5 × 10^5^) groups exhibited similar, uniformly distributed mineralized tissue coverage throughout the defects, but the coverage was lower than that of the BMP implant group [102]. In addition, robust expression of dentin and bone differentiation marker expression was observed in human DPSC-seeded scaffolds, whereas, in contrast, BMP and scaffold-alone implants exhibited only bone and not dentin differentiation marker expression [102]. Human DPSCs/HUVECs seeded on acellular tyrosine-derived polycarbonate E1001(1K)/β-TCP constructs were implanted into rabbit craniomaxillofacial (CMF) bone defects at 1 and 3 months. The results showed that human DPSC-seeded E1001(1K)/β-TCP constructs support the formation of osteodentin-like mineralized jawbone tissue closely resembling that of a natural rabbit jawbone. Although unseeded scaffolds supported limited alveolar bone regeneration, more robust and homogeneous bone formation was observed in the hDPSC/HUVEC-seeded constructs, suggesting that human DPSCs/HUVECs contributed to enhanced bone formation. Further, the bioengineered jaw bone recapitulated the typical morphology of natural rabbit jawbone, was highly vascularized, and exhibited active remodeling, as evidenced by the presence of osteoblasts and osteoclasts on newly formed bone surfaces [103].

Li Y et al., isolated DPSCs from autologous inflammatory dental pulp from two patients, loaded these onto a scaffold of β-tricalcium phosphate, and then engrafted the construct into the periodontal defective area in the root furcation. Clinical and radiographic evaluation showed that DPSCs from inflammatory dental pulp tissues were able to graft and regenerate new bone to repair periodontal defects 9 months after surgical reconstruction [104]. Hu J et al., isolated human DPSCs and used the DPSCs as cell sheets or single cell injections to treat miniature pig periodontitis with bone defects (5 mm in width, 7 mm in length, and 3 mm in depth). After 12 weeks, both the human DPSC sheet treatment and human DPSC injection significantly improved periodontal tissue healing clinically in comparison with the control group [105]. The volume of regenerated bone in the human DPSC cell sheet group (52.7 ± 4.1 mm^3^) was significantly larger than in the human DPSC injection group (32.4 ± 5.1 mm^3^) (*p* < 0.05). The percentage of bone/total volume in the periodontium in the human DPSC injection group was 12.8 ± 4.4%, while it was 17.4 ± 5.3% in the human DPSC sheet group (*p* < 0.05). This indicated that the transplantation of human DPSCs into this large animal model significantly improved periodontal bone regeneration and soft tissue healing [105]. Lyu J et al., compared the recombinant peptide Cellnest™ 3D stem cell matrix (CellSaic) containing rat DPSCs and BMMSCs for rat congenital cleft fracture repair. Cultured CellSaic in osteoinductive media generated more mineralized tissues than the control group without osteoinductive media. Overall, rat BMSC-CellSaic and rat DPSC-CellSaic made with Cellnest™ as a scaffold provided excellent support for promoting bone regeneration in rat mandibular congenital defects [106]. Both differentiated and undifferentiated rat DPSC-CellSaic, but only differentiated rat BMSC-CellSaic, could induce the formation of new bone tissue. Rat DPSC-CellSaic represents a better source for craniofacial bone defect repair than rat BMSC-CellSaic [106]. Another study evaluated the use of ceramic nanocomposites of hydroxyapatite/titania/calcium silicate (C1, C2, and C3) with hDPSCs, demonstrating enhancement in bone healing and osteointegration in a rabbit tibia defect model compared to the control group [107].

A single-center, double-blind, randomized, split-mouth, and controlled clinical trial was conducted to evaluate the beneficial effects of uncultured DPSCs delivered in a collagen matrix on the post-extraction sockets of impacted mandibular third molars in 32 patients. The clinical, radiological, and surgical characteristics of the impacted third molars in both the control and experimental groups were homogeneous. No significant differences were observed in terms of bone repair when analyzing the density (*p* = 0.4203, neuroradiologist 1; *p* = 0.2525, neuroradiologist 2) or interdental septum height (*p* = 0.2280, neuroradiologist 1; *p* = 0.4784, neuroradiologist 2). The study could not demonstrate that autologous dental pulp mesenchymal stem cells reduced socket bone resorption following the extraction of inferior third molar extraction [108].

### 5.2. DPSCs Modified with Different Genes for Bone Tissue Engineering

Song F et al., discovered that Pannexin3 (Panx3) was upregulated in a time-dependent manner during the osteogenic differentiation of DPSCs. The overexpression of Panx3 promoted osteogenic differentiation of hDPSCs, as evidenced by the upregulated expression of mineralization-related markers, increased ALP activity, and enhanced ALP and Alizarin red staining. Conversely, the depletion of Panx3 resulted in impaired osteogenic differentiation [109]. Panx3 was found to interact with the Wnt/β-catenin signaling pathway, forming a negative feedback loop. However, Wnt/β-catenin did not contribute to the enhancement of osteogenic differentiation as observed in Panx3 overexpression. Moreover, Panx3 promoted osteogenic differentiation of human DPSCs by increasing the ERK signaling pathway. In vivo, micro-CT and histological staining results showed that Panx3-modified human DPSCs significantly improved ossification of critical-size bone defects [109].

Song D et al., utilized DPSCs transduced with Adeno-SIRT1 to enhance distraction osteogenesis in a rabbit tibia model. The authors demonstrated that the Ad-SIRT1 overexpressing hDPSC group exhibited improved bone formation, a higher bone mineral density (BMD), and increased bone mineral content (BMC) compared to the AD-GFP-DPSCs and no cell groups [110]. Wang W et al., revealed that ephrinB2 was upregulated following the osteogenic induction of human DPSCs. The overexpression of ephrinB2 enhanced the osteogenic differentiation capacity of human DPSCs in vitro. Additionally, p-ephrinB2 was upregulated by ephrinB2 overexpression [111]. In a canine bone defect model, Lenti-ephrinB2 transduced canine DPSCs embedded in PuraMatrix Peptide Hydrogel and significantly improved the quality of newly regenerated alveolar bone, as demonstrated by the higher trabecular bone volume per tissue volume (BV/TV), trabecular thickness, and radiographic analysis [111]. Furthermore, Ets variant 2 (ETV2) transcription factor-transduced DPSCs exhibited enhanced osteogenesis in vitro compared to un-transduced human PDSCs. The transplantation of ETV2-transduced DPSCs using a β-TCP scaffold also demonstrated increased bone formation in both a rat calvarial defect model and a nude mice ectopic bone formation model [112].

### 5.3. DPSCs Treated with Small Molecule or Its Inhibitor Enhance Bone Repair

DPSCs were harvested from six healthy patients aged 18–29 years and cultured in normal medium (NM), osteogenic medium (OM), or OM with a helioxanthin derivative, 4-(4-methoxyphenyl) pyrido[40,30:4,5]thieno[2,3-b]pyridine-2-carboxamide (TH). These cells were then fabricated into cell sheets and labeled with PKH26. After transplantation into mouse tibial fractures, it was demonstrated that the transplanted OM+TH-treated DPSC sheets localized to the fracture site and facilitated bone formation [113]. Human DPSCs treated with chrysin, a flavanol extracted from oroxylum seeds, exhibited increased osteogenic differentiation in vitro and enhanced β-TCP-induced mineralization in a mouse model of heterotopic bone formation and a rat calvaria defect model by upregulating SMAD3 [114]. DPSCs treated with melatonin showed enhanced osteogenic differentiation by increasing P38MAPK activity. In vivo, the transplantation of the DPSCs in a calvarial defect model, using an MBCP scaffold, resulted in improved bone regeneration compared to just the scaffold or an empty control, though it was not significantly better than untreated DPSCs [115].

Combining sclerostin antibody systemic injection with DPSC implantation with collagen hydrogel increased bone regeneration in WT mice. On the other hand, the implantation of DPSCs isolated from SOSTKO mice exhibited similar bone regeneration in WT mice compared to the bone regeneration in SOSTKO mice, indicating that the downregulation of SOST expression (Wnt signaling inhibitor) in DPSCs is also important to enhance their bone regeneration potential [116].

### 5.4. DPSC or PDLSC Exosomes for Bone Tissue Engineering

Healthy human periodontal ligament stem cell (PDLSC)-derived exosomes, loaded with hydrogel or β-TCP, accelerated bone formation in alveolar bone defects in rat models of periodontitis-induced bone loss. Mechanistically, human PDLSC exosomes suppressed the over-activation of canonical Wnt signaling and restored the osteogenic differentiation capacity of inflammatory PDLSCs [117]. Human PDLSC-derived sEVs (P-EVs) have been shown to enhance the proliferation and migration of BMMSCs through increased phosphorylation of AKT and extracellular signal-regulated kinase 1/2 (ERK1/2). The role of P-EV-induced adenosine receptor signaling in AKT and ERK1/2 phosphorylation was a key mediator contributing to enhanced BMMSC proliferation and migration. In vivo, P-EV/Matrigel accelerated bone tissue repair by increasing cell infiltration compared to the control group. Additionally, exosomes derived from PDLSCs enhanced alveolar bone defect healing in a rat model [118].

Taken together, the above studies provide evidence that stem cells derived from dental tissues may be another source of stem cells for bone tissue engineering, especially in the field of craniofacial bone regeneration. These studies are summarized in Supplemental Table S1.

## 6. Periosteal Stem Cells (PSCs)

The periosteum is indispensable in bone repair and an important source of skeletal stem cells (SSCs) for endogenous bone regeneration [119]. However, relatively few studies have used isolated PSCs for the purpose of bone regeneration and tissue engineering.

### 6.1. PSCs Alone or Combined with Bone Growth Factors for Bone Tissue Engineering

Van Gastel N et al., demonstrated that murine PSCs, pretreated with FGF2, resulted in the complete healing of large bone defects in mouse femora via endochondral bone healing [120]. Ji W et al., compared human PSCs seeded with different doses of BMP6 or various clinical grades of calcium phosphate scaffold (ChronOs^®^, ReproBone™, and CopiOs^®^) for bone regeneration in an ectopic bone formation model. They reported that cells seeded on CaP scaffolds with an intermediate Ca2^+^ release rate, combined with low or medium dosages of BMP6 (810 ng and 3240 ng BMP6/scaffold), demonstrated robust new bone formation at 5 weeks, and the new bone was derived from both donor and host cells [121]. PSCs transplanted into 3 cm long fresh tibia defects in sheep showed similar effects as BMP6 or BMP2 protein treatment. However, in a 4.5 cm biologically exhausted tibial defect, only PSCs in combination with the BMP group promoted bone defect healing in sheep compared with other groups. This result indicated that exogenous PSCs are very important when host cells are compromised [122]. To determine the role of HIF-1α in PSC-mediated bone regeneration, it was found that knockdown HIF-1α impaired the bone regeneration and osteogenesis of PSCs both in vivo and in vitro. Furthermore, the knockdown of HIF-1α also reduced periostin (POSTN) expression, and the addition of recombinant POSTN partially rescued the osteogenic inhibition. The phosphorylation levels of PI3K and AKT were enhanced with HIF-1α overexpression and inhibited when HIF-1α was knocked down; the addition of a PI3K or AKT activator could partially rescue POSTN expression [123].

### 6.2. PSCs from Different Anatomic Origins Demonstrate Variable Bone Regeneration Capacities

Human PSCs isolated from different anatomic origins (tibia, maxilla, and mandible) exhibited similar trilineage differentiation in vitro. However, for in vivo bone formation, 8 weeks after ectopic implantation in nude mice, it was observed that constructs seeded with tibial and mandibular human PSCs, but not maxilla PSCs, regenerated bone effectively [124]. Tang Y et al., investigated the differences in PSCs from the mandible and femur and their potential responses to YAP signaling. Mandibular PSCs were cubic-shaped with better proliferation, while femoral PSCs were slender with reduced cell viability compared to mandibular PSCs. Mandibular PSCs outperformed femoral PSCs in their ALP activity, osteogenic-related genes’ mRNA expression, and calcium deposition at a later stage. Interestingly, the downregulation of YAP enhanced the ALP activity, the related genes’ mRNA expression, and the calcium deposits of femoral PSCs, while inhibiting those characteristics in mandibular PSCs in vitro. Mandibular PSCs also demonstrated superior bone repair in both mandible and femoral defect models, likely due to their different embryonic origins and modes of bone formation [125].

### 6.3. PSC Secretomes for Bone Tissue Engineering

Pranskunas M et al., evaluated the functionality of the secretome isolated from PSCs in basal or osteogenic-induced conditions in healing critical-size calvarial bone defects in a rabbit model using a bioceramic xenograft scaffold. The osteogenic-induced PSC secretome showed increased diversity of proteins, especially those related to osteogenesis [126]. Micro-CT and histological morphometric analysis revealed that bioceramic xenografts implanted with secretomes enhanced the new bone formation process, with the osteogenic-induced secretome promoting the greatest bone tissue formation. Therefore, the application of the PSC secretome, particularly from osteogenic-induced PSCs, may be an effective therapeutic approach to enhancing bone tissue healing and regeneration [126].

In summary, PSCs isolated from different origins or their secretome can promote bone repair when combined with different scaffolds or factors and are summarized in Supplemental Table S1.

## 7. Amniotic Fluid-Derived Stem Cells (AFDSCs)

Human AFDSCs represent a unique population of stem cells that are easily accessible and characterized as an intermediate stage between embryonic stem cells (ESCs) and adult stem cells. Human AFDSCs can be induced towards osteoblastic differentiation by rhBMP7 and respond more strongly to rhBMP7 than human BMMSCs. When these AFDSCs were then seeded on nanofibrous scaffolds (NF scaffolds) with a morphology that is similar to that of natural collagen fibers, they exhibited significantly enhanced ALP activity, calcium content, and von Kossa staining and greater expression of osteogenic genes than those on the traditional scaffolds (i.e., solid-walled scaffolds) both in vitro and in newly regenerated bone in vivo [127]. Human AFDSCs have been shown to regenerate bone in critical-size calvarial bone defects via direct differentiation into osteogenic and endothelial cell lineages [100]. They also undergo in vitro osteogenic differentiation when seeded onto poly(lactide-co-glycolide) (PLGA)–bladder submucosa matrix (BSM) composite scaffolds [128]. Mohammed EEA et al., reported that human second-trimester AFDSCs, when seeded on a 30% nano-hydroxyapatite chitosan scaffold, underwent osteogenic differentiation in vitro. When transplanted into a rabbit tibia defect, they enhanced bone formation, exhibiting complete bone defect healing at 4 weeks after surgery [129]. Using human AFDSCs to fabricate osteogenic and vascular cell sheets and applying these sheets to a rat 4 mm calvarial bone defect enhanced bone regeneration more than osteogenic or vascular cell sheets alone, and more than the control group [130].

Wang M reported that rat AFDSCs exhibit typical fibroblastoid morphology, stable proliferation activity, and multi-differentiation potential. Flow cytometry analysis demonstrated that these cells were positive for CD29, CD44, and CD90, while negative for hematopoietic markers such as CD34 and CD45. These cells, when premixed with a platelet-rich plasma (PRP) gel, demonstrated superior regenerative capacity in restoring alveolar bone defects, as evidenced by micro-CT and histological analyses at 4 and 8 weeks post-surgery, compared to control groups. Moreover, the implanted AFDSCs survived in the defect site and directly participated in the bone tissue regeneration [131]. Ghaffarinovin Z et al., isolated rat AFDSCs and seeded them onto random polycaprolactone (PCL) fibrous scaffolds combined with PRP, applying this combination to repair calvarial bone defects. The authors found that collagen type I was expressed by AFDSCs cultured on the scaffold. Adding PRP promoted the formation of blood vessels and collagen type I expression in the defective area [132].

These studies indicated that AFDSCs are another source of stem cells for bone tissue engineering (Supplemental Table S1).

## 8. Peripheral Blood-Derived Mesenchymal Stem Cells (PBMSCs)

Recently, new methods have been investigated to isolate MSCs from peripheral blood due to their relatively non-invasive, easily accessible nature compared to other sources such as bone marrow. The Gang Li group first isolated PBMSCs from rabbit blood, comparing their bone regeneration capabilities with rabbit BMMSCs in a rabbit ulna 20 mm defect model. The authors demonstrated that rabbit PBMSCs possessed multi-differentiation potential that is comparable to that of BMMSCs [133]. Allogenic PBMSCs seeded onto a porous calcium phosphate resorbable scaffold enhanced bone regeneration in the rabbit ulna critical-size bone defect model, suggesting that allogenic PBMSCs might be a new source of circulating osteogenic stem cells for bone regeneration and tissue engineering [133]. Zheng RC et al., also isolated PBMSCs from rabbits, showing that PBMSCs had a similar proliferation rate, as indicated by the BrdU-positive cells, compared to BMMSCs. PBMSCs were positive for CD90 but negative for CD14. They exhibited osteogenic, adipogenic, and chondrogenic differentiation in vitro and bone formation in vivo in an immunocompromised mouse root canal model. Further histological results demonstrated that the PBMSC and BMMSC groups showed more newly formed bone than the HA/TCP and defect groups in the upper and lower chambers at 6 weeks, as well as in the upper canal at 3 weeks; however, there was no difference in newly formed bone among all groups in the lower canal at 3 weeks. The PBMSCs exhibited characteristics and bone-regenerative capacity that were similar to those of BMMSCs both in vitro and in vivo [134]. Chen L et al., developed a three-dimensional (3D) co-culture system using a biphasic calcium phosphate bioceramic (BCPB) scaffold seeded with rabbit PBMSCs and endothelial progenitor cells (EPCs) to improve new bone formation and vascularization for long bone segmental defects. The results showed that both osteogenic and vascular-related genes were upregulated when EPCs were co-cultured with PBMSCs. In addition, BCPB is biocompatible, and the expression levels of osteogenic and vascular-related markers were also upregulated in the 3D co-culture system [135]. The implantation of seeded PBMSCs and EPCs within a modified BCPB resulted in substantial new bone formation and promoted vascularization in a rabbit large bone defect model [135]. Wang H et al., used a similar concept to construct a novel vascularized tissue-engineered bone (VTEB) by using rabbit PBMSCs and peripheral blood EPCs (PBEPCs) seeded on a 3D-printed biphasic calcium phosphate (BCP) scaffold with a highly bioactive nano-hydroxyapatite (nHA) coating (nHA/BCP). They tested it in a rabbit femoral segmental bone defect (SBD) model. In vivo, it was found that among the four groups (BCP, BCP-PBEPC/PBMSC, nHA/BCP, and nHA/BCP-PBEPC/PBMSC), the nHA/BCP-PBEPC/PBMSC group induced the best formation of blood vessels and newly formed bone and the best repair of the SBD. Therefore, taking advantage of the synergistic effect of nHA and PBEPC/PBMSC on angiogenesis and osteogenesis in the BCP scaffold is a new strategy for efficient bone repair [136].

Li S et al., isolated a subset of CD45^−^ cells with fibroblast-like morphology from mouse peripheral blood. These cells were adherent to plastic; negative for CD34, CD19, CD11b, lineage, and c-kit; and positive for Sca-1, CD73, CD44, CD90.1, CD29, CD105, CD106, and CD140a. These cells exhibited osteogenic, chondrogenic, and adipogenic differentiation potential and induced the healing of critical-size calvarial bone defects when using hydroxyapatite-poly(lactic-coglycolic acid) (HA-PLGA) scaffolds in vivo [137].

Human PBMSCs were also isolated from peripheral blood. Purified populations of PBMSCs can be obtained within a short period of time using this protocol, with a success rate of 60%. Human PBMSCs cultured under hypoxia possessed potent multilineage differentiation capacity. They also expressed Nanog and Lgr5, as well as a series of MSC surface antigens (including CD29, CD90, CD105, and CD73). Additionally, using an ectopic bone formation model, it was demonstrated that the transplantation of human PBMSCs regenerate bone using a porous and resorbable β-tricalcium phosphate (β-TCP) scaffold in vivo [138].

These studies demonstrate that PBMSCs represent a potentially alternative cell source in the treatment of large bone defects, and the studies are summarized in Supplemental Table S1.

## 9. Umbilical Cord-Derived Mesenchymal Stem Cells (UC-MSCs)

Human umbilical cords represent another source of stem cells that can be banked and used for both mother and infant.

### 9.1. UC-MSCs Delivered with Different Scaffolds for Bone Tissue Engineering

Diao Y et al., demonstrated that human umbilical cord-derived mesenchymal stem cells (UC-MSCs) at the fourth passage were positive for CD29, CD44, CD71, CD73, CD90, and CD105 and negative for CD14, CD34, CD45, and CD117. Furthermore, these cells expressed HLA-A, B, and C (MHC-I), but not HLA-DP, DQ, DR (MHC-II), or costimulatory molecules such as CD80 and CD86. Following incubation in specific inductive media for 3 weeks, the cultured cells were shown to possess the potential to differentiate into adipogenic, osteogenic, or chondrogenic lineages in vitro [139]. When UC-MSCs loaded with a biomimetic artificial bone scaffold material were implanted subcutaneously into the back of Balb/c nude mice for four to twelve weeks, osteogenesis was observed in vivo [139]. Human UC-MSCs transfected with the pEGFP-OSX plasmid showed enhanced in vitro osteogenic differentiation, and co-delivery using a PLGA scaffold promoted bone formation in nude mice 4 weeks after transplantation [140].

A retrospective analysis of the clinical effects (Randomized Clinical Trials) of transplanted human UC-MSCs for the treatment of osteonecrosis of the femoral head (ONFH) demonstrated that human UC-MSCs grafted by intra-arterial infusion organized effective perfusion of the femur head, as demonstrated by an increase in the oxygen delivery index (ODI) at 3 days post-operation. The MRI results revealed that at 12 and 24 months after treatment, the necrotic volume of the femoral heads was significantly reduced, and no obvious abnormalities were observed. These data indicate that intra-arterially infused human UC-MSCs migrate into the necrotic field of femoral heads and differentiate into osteoblasts, thus improving the avascular necrosis of femoral heads. This finding suggests that the intra-arterial infusion of human UC-MSCs is a feasible and relatively safe method for the treatment of femoral head necrosis [141]. Mesenchymal stem cells derived from the human umbilical cord (Wharton’s jelly (WJ-MSC)) and seeded onto a Bio-Oss^®^ scaffold can differentiate into osteoblast-like cells, and when injected, the Bio-Oss^®^ scaffold significantly enhances calvarial defect healing in a rat model [142]. Others have shown that miR-196a-5p could repress proliferation and stimulate osteogenic differentiation and WJCMSC sheet-derived ECM deposition, thus promoting new bone formation and rat calvarial bone defect closure. Furthermore, SERPINB2 is a key downstream gene that is involved in the miR-196a-5p-promoted WJCMSC osteogenesis [143].

### 9.2. UC-MSC-Derived Exosomes for Bone Tissue Engineering

Human UC-MSC-derived exosomes could effectively promote the proliferation, migration, and osteogenic differentiation of a murine calvariae pre-osteoblast cell line in vitro. When an injectable hydroxyapatite (HAP)-embedded in situ crosslinked hyaluronic acid–alginate (HA-ALG) hydrogel system was used with the exosome, the combination significantly enhanced bone regeneration in rats [144]. Human UM-MSC-derived extracellular vesicles (UC-MSC-EVs) have a size ranging from 60 nm to 150 nm and express CD9, CD63, CD81, and TSG101. The systemic administration of human UC-MSC-EVs prevented bone loss and maintained bone strength in osteoporotic mice by enhancing bone formation, reducing marrow fat accumulation, and decreasing bone resorption. The beneficial effect is thought to correlate with highly expressed levels of a pro-osteogenic protein, C-type lectin domain family 11, member A (CLEC11A), in human UC-MSC-EVs. In addition, human UC-MSC-EVs enhanced the shift from adipogenic to osteogenic differentiation of BMMSCs by delivering CLEC11A in vitro [145].

Another study showed that UC-MSC-derived exosomes (UC-MSC-EXOs) encapsulated in hyaluronic acid hydrogel (HA-Gel) and combined with customized nano-hydroxyapatite/poly-ε-caprolactone (nHP) scaffolds markedly enhanced bone regeneration in vivo. Moreover, the in vitro results demonstrated that UC-MSC-EXOs promoted the proliferation, migration, and angiogenic differentiation of endothelial progenitor cells (EPCs) but did not significantly affect the osteogenic differentiation of BMMSCs [146]. Importantly, mechanistic studies revealed that exosomal miR-21 was the potential intercellular messenger that promoted angiogenesis by upregulating the NOTCH1/DLL4 pathway [146]. A human UC-MSC-derived exosome-loaded chitosan/hydroxyapatite (CS/HA) scaffold also regenerated significantly more bone than the scaffold-only group or the control group in a rat calvarial bone defect model [147].

Taken together, these studies demonstrated that UC-MSCs and their exosomes combined with scaffolds are another choice for bone tissue engineering, and the studies are summarized in Supplemental Table S1.

## 10. Urine-Derived Stem Cells (UDSCs)

### 10.1. UDSCs Loaded with Different Scaffold Materials for Bone Tissue Engineering

Guan et al., isolated human UDSCs via a simple centrifuge method to obtain cell pellets that were adherent to plastic. The human UDSCs demonstrated multipotent differentiation potentials and expressed CD29, CD44, CD73, and CD90 and were negative for CD34, CD45, CD133, and HLA-DR. When these human UDSCs were loaded with β-TCP, they induced substantial new bone formation and subsequent healing of segmental bone defects in rat femora [148]. Human UDSCs transduced with Lenti-BMP2 showed enhanced osteogenic differentiation capacity and enhanced ectopic bone formation in nude mice using a β-TCP scaffold, and the direct contribution of human cells was observed in the newly regenerated bone [149]. Human UDSCs seeded on calcium silicate (CS) particles incorporated into poly (lactic-co-glycolic acid) (PLGA) composite scaffolds showed enhanced cell proliferation, ALP activity, calcium deposition, and expression of certain osteoblast-related genes and proteins via inducing the Wnt/β-catenin signaling pathway. Furthermore, human UDSCs seeded on the CS/PLGA scaffold promoted bone formation in vivo using a muscle ectopic bone formation model in immune-compromised mice [150].

Others also showed that human UDSCs loaded with surface-mineralized biphasic calcium phosphate ceramics (BCPs) significantly promoted bone defect healing in a New Zealand white rabbit ulna segmental bone defect model [151]. Another study demonstrated that a graphene oxide–modified silk fibroin/nano-hydroxyapatite scaffold loaded with human UDSCs significantly promoted rat calvarial bone defect healing via modulation of the polarization of macrophages to the M2 macrophage phenotype that promotes bone regeneration [152]. Liu M et al., combined human UDSCs with a biphasic calcium phosphate (BCP) bioceramic ornamented with chitosan sponges (CSs) (CS/BCP) hybrid scaffold to construct tissue-engineered bone and evaluated whether the combination promotes bone regeneration in large ulna segmental bone defects in rabbits. The results demonstrated that human UDSCs can differentiate into osteoblasts, and the human UDSCs adhered, proliferated, and differentiated on CS/BCP hybrid scaffolds. Micro-CT, biomechanical detection, and histological analyses revealed that the combination of human UDSCs and the CS/BCP hybrid scaffold enhanced bone regeneration more effectively compared with conventional pure BCP scaffolds, indicating that human UDSCs can be used as a cell source for bone tissue engineering [153]. Wu S et al., reported an injectable BMP2-releasing chitosan microsphere/type I collagen hydrogel (BMP2-CSM/Col I hydrogel) loaded with human UDSCs for bone regeneration. The results showed that human UDSCs proliferated in a time-dependent fashion, spread with good extension, and interconnected with each other in different hydrogels both for 2D and 3D models. Sustained released BMP2 increased the ALP activities and mineral depositions of UDSCs in a 2D culture and enhanced the expression of osteogenic genes and proteins in 3D culture [154]. In vivo, the mixture of human UDSCs and BMP2-CSM/Col I hydrogels effectively enhanced bone regeneration in a rat calvarial bone defect model, and the ratio of new bone volume to total bone volume was 38% after 8 weeks of implantation, although the defect was not completely healed. Further, human UDSCs differentiated into osteoblasts in the newly regenerated bone, as demonstrated by the human nuclear-positive staining [154].

Xing F et al., fabricated a 3D-printed poly(ε-caprolactone) (PCL) scaffold with a nano-topographical surface and loaded it with human UDSCs for bone regeneration. The topological 3D-printed PCL scaffolds (TPSs), fabricated by surface epiphytic crystallization, possessed uniformly patterned nanoridges, with an element composition and functional grouping of nanoridges that were the same as those of the native PCL. Compared with bare 3D-printed PCL scaffolds (BPSs), TPSs have a higher ability for protein adsorption and mineralization in vitro [155]. The proliferation, cell length, and osteogenic gene expression of UDSCs on the surface of TPSs were significantly higher than that of BPSs. TPSs loaded with UDSCs exhibited enhanced bone regeneration in rabbit cranial bone defects when compared to the BPS/UDSC group and the scaffold-only group, but the newly regenerated bone appeared as mesh due to the scaffold not being absorbed [155].

Zhang X et al., fabricated a 3D-printed polylactic acid and hydroxyapatite (PLA/HA) composite scaffold with human UDSCs to study its therapeutic effect in a rat model of skull defects (5 mm). Human UDSCs were inoculated onto PLA/HA and PLA scaffolds using 3D printing and implanted into a 5 mm calvarial bone defect of Sprague Dawley rats. The results demonstrated that the PLA/HA scaffold loaded with human UDSCs effectively promoted new bone regeneration in the defective area [156]. Micro-CT images showed that in the PLA/HA/cell group, the defective area was almost entirely covered by newly formed bone (coverage of 96.7 ± 1.6%), and the coverage was greater than that in the PLA cell group (coverage of 74.6 ± 1.9%) at 12 weeks [156].

### 10.2. UDSC Exosomes for Bone Tissue Engineering

Li H et al., reported that human UDSC-derived extracellular vesicles (UDSC-EVs) exhibited a cup-like morphology with a double-layered membrane structure, which was positive for CD63 and TSG101 and negative for calnexin. In vitro, UDSC-EVs promoted the osteogenic differentiation of BMMSCs and reduced proinflammatory factor production, as well as osteoclastic activity, in RAW264.7 macrophage cells [157]. In vivo, local injection of UDSC-EVs around the central sites of the calvaria of ultra-high-molecular-weight polyethylene (UHMWPE) particle-induced osteolysis decreased inflammatory cytokine generation and osteolysis compared with the control groups and significantly increased bone formation [157].

Human UDSC-derived exosomes delivered with Gelatin methacrylate (GelMA) and hyaluronic acid methacrylate (HAMA)/nano-hydroxyapatite (nHAP) hydrogels could promote the osteogenesis of bone marrow mesenchymal stem cells (BMMSCs) and the angiogenesis of EPCs in vitro [158]. Additionally, the in vivo results demonstrated that this composite hydrogel could significantly promote the defect repair of cranial bone in the rat model. It was also found that a UDSC-EXOs/GelMA-HAMA/nHAP composite hydrogel can promote the formation of H-type vessels in the bone regeneration area, enhancing the therapeutic effect of bone regeneration [158].

In summary, these human UDSC studies demonstrated the promise of clinical translation for bone tissue engineering and are summarized in Supplemental Table S1.

## 11. Stem Cells from the Apical Papilla (SCAP)

Sonoyama W et al., first isolated SCAP from the human root apical papilla and demonstrated that SCAP exhibits higher proliferation, mineralization, migration, and telomere activity and multipotent differentiation. Using a minipig model, it was demonstrated that both human SCAP and periodontal ligament stem cells (PDLSCs) generated a root/periodontal complex that was capable of supporting a porcelain crown, resulting in normal tooth function [159,160].

### 11.1. SCAP for Bone and Dental Tissue Engineering

Human SCAPs treated with IGF1 are more osteogenic and less prone to odontogenic differentiation; when transplanted ectopically in the renal capsule, IGF1-treated SCAPs mostly gave rise to bone-like tissues, while untreated SCAPs mainly generated dentin–pulp-complex-like structures after transplantation into immunocompromised mice [161]. Zhang J et al., presented immortalized murine SCAP (iSCAP) with a reversible immortalization system expressing SV40 T and flanked with Cre/loxP sites. In these iSCAPs, BMP9 upregulates Runx2, Sox9, and PPARγ2 and odontoblastic markers and induces alkaline phosphatase activity and matrix mineralization. The in vivo stem cell implantation experiment indicated that iSCAPs could differentiate into bone, cartilage, and, to a lesser extent, adipocytes, upon BMP9 stimulation [162]. Zhang HM et al., reported that murine SCAPs’ osteogenic differentiation was synergistically regulated by BMP9 and Wnt signaling. In vivo, BMP9-transduced iSCAPs induced robust ectopic bone formation; murine iSCAPs stimulated with both BMP9 and Wnt3A exhibited more mature and highly mineralized trabecular bone formation [163]. Wang W et al., demonstrated that human SCAP treated with exhibited increased osteogenic differentiation and dentin sialophosphoprotein accumulation both in a monolayer culture and on 3D PLLA nanofibrous microspheres (NF-MS) spinner flask cultures in vitro. Using NF-MS-controlled BMP2 release combined with SCAPs promoted more mineralization and osteodentin formation compared to a BSA-releasing control in a dose-dependent and time-dependent manner [164]. SCAPs isolated from beagle dogs were resuspended in peripheral blood and implanted in a dog periapical periodontitis model with infection eliminated. The newly formed tissues were much thicker compared to those in the endogenous blood of the peripheral blood-filled root canal and showed a dentine tubule like structure instead of bone lacunae, although the orientations of these tubules were varied [165]. Li G et al., performed local injections of human SCAPs in an experimental periodontitis model using miniature pigs. Local SCAP injections significantly increased alveolar bone regeneration than in the saline-injected group, as revealed by clinical assessment, microCT, and histology [166]. Yang H et al., demonstrated that distal-less homeobox 5 (DLX5) enhanced the osteo/dentino-genesis capabilities of human SCAPs and the in vivo via upregulation of lysine-specific demethylase 4B (KDM4B) [167]. Xiao M demonstrated that culturing human SCAPs on a polysaccharide hydrogel (Vitro-Gel 3D) demonstrated favorable viability and proliferation in vitro. SDF-1α and BMP-2 cotreatment of SCAPs enhanced odontogenic differentiation-related gene and protein expression and promoted odontogenic differentiation of SCAPs in vivo [168]. Locally injected human SCAPs overexpressing SFRP2 also significantly enhanced new bone formation in a periodontitis model in miniature pigs [169]. Deng J et al., demonstrated that transduced human SCAPs with lenti-GFP/PDGFBB increased cell proliferation. When seeded into thermosensitive hydrogel and transplanted into a calvarial defect of Sprague Dawley^®^ (SD)-rats, they increased bone formation in the defect, with significantly higher defect coverage than other groups [170].

### 11.2. Exosomes from SCAPs for Bone and Dental Tissue Engineering

Zhuang X et al., demonstrated that when human SCAP-derived exosomes (SCAP-Exo) were introduced into the root fragment containing bone marrow mesenchymal stem cells (BMMSCs) and transplanted subcutaneously into immunodeficient mice, dental pulp-like tissues and the newly formed dentine were deposited onto the existing dentine in the root canal. SCAP-Exo-treated BMMSCs significantly increased the gene and protein expression of dentine sialophosphoprotein and mineralized nodule formation [171]. Jing X et al., reported that SCAP-Exo facilitated angiogenesis and osteogenesis both in normal or diabetic conditions. They further designed a bioresponsive polyethylene glycol (PEG)/DNA hybrid hydrogel that can be triggered by the elevated pathological cue (MMP-9) in response to the dynamic diabetic microenvironment. They demonstrated that the administration of the injectable SCAP-Exo-loaded PEG/DNA hybrid hydrogel into the mandibular bone defect of diabetic rats promoted vascularized bone regeneration. This positive effect of SCAP-exo correlated with highly expressed miRNA-126-5p and miRNA-150-5p [172]. Zhang T et al., used non-invasive low-intensity pulsed ultrasound (LIPUS) as the stimulation for improving both oral SCAP extracellular vesicle (SCAP-EV) production and effectiveness and demonstrated that human SCAP had intensity-dependent, pro-osteogenic, and anti-inflammatory responses to LIPUS without significant cytotoxicity or apoptosis. The stimuli increased the secretion of EVs by promoting the expression of neutral sphingomyelinases in SCAP. In addition, EVs from LIPUS-induced SCAPs exhibited stronger efficacy in promoting the osteogenic differentiation and anti-inflammation of periodontal ligament cells and alleviating oral inflammatory bone loss in vivo via increased miR-935 [173].

Taken together, SCAP cells are a promising cell source for craniofacial and dental bone regeneration and repair (Supplemental Table S1).

## 12. iPSC-Derived MSCs or Osteoblasts for Bone Tissue Engineering

iPSCs generated by reprogramming somatic cells using four transcription factors (Oct4, NANOG, SOX-2, and C-Myc) or KL4, possess pluripotency, can differentiate into virtually any kind of functional cells, offering great potential for tissue regeneration such as bone tissue engineering [174,175,176,177,178]. In this section, we review the therapeutic application of iPSC-derived MSCs or osteoblasts for bone tissue engineering.

### 12.1. iPSC-Derived MSCs for Bone Tissue Engineering Using Different Scaffolds

In 2011, Jake Chen’s group overexpressed the SATB homeobox 2 (SATB2) gene in mouse iPSCs. They found that SATB2-overexpressed iPSCs also expressed higher levels of osteogenic markers. When combining SATB2 iPSCS with silk scaffolds and transplanting them into critical-size calvarial bone defects created in nude mice, enhanced new bone formation was observed in the calvarial defects compared to vector-transduced iPSCs and other control groups [179]. Hong SG et al., using non-human primate iPSCs for bone regeneration, found that undifferentiated autologous iPSCs formed mature teratomas in a dose-dependent manner, noting that the tumor formation was accompanied by an inflammatory reaction. However, iPSC-derived mesodermal stromal-like cells formed new bone in vivo without any evidence of teratoma formation [180]. Xie J et al., demonstrated that mouse iPSC-derived MSCs (iPSCs-MSC) seeded on biomimetic nanofibers of hydroxyapatite/collagen/chitosan (HAp/Col/CTS)HAp/Col/CTS scaffolds enhanced osteogenic differentiation and increased bone regeneration 2-fold compared with other scaffold groups in a calvarial bone defect model [181]. Sheyn D et al., using short-term exposure of human iPSCs’ embryonic bodies to TGFβ1 to induce iPSCs-MSCs, successfully generated early iMSCs (aiMSCs) and late iMSCs (tiMSCs) that both possess multipotent differentiation capacities. However, when BMP6-overexpressing tiMSCS (plasmid-mediated overexpression) were injected in a muscle ectopic bone model, the tiMSCs generated very little bone, while BMP6-overexpressing aiMSCs generated a substantial amount of new bone. Interestingly, when both populations of BMP6-overexpressing iMSCs were transplanted into rabbit non-union radial bone defects using collagen type I biodegradable scaffolds, they both repaired bone as effectively as BMMSCs at 8 weeks [182]. Zhang M et al., developed a 3D rotary culture system to rapidly differentiate iPSCs to the chondrogenic mesoderm lineage using BMP4 and FGF2. The cartilage pellets that were produced using these iPSCs derived mesoderm cell regenerated cartilage in an osteochondral bone defect, as well as vascularized bone, using a rat calvarial bone defect without the need for any scaffold [183]. Chien KH et al., reported that seeding BMP6-treated rat iPSCs into chitosan/gelatin/glycerol phosphate hydrogels and then implanting them into maxillary–molar defects increased the bone volume, trabecular number (Tb.N), and trabecular thickness (Tb.Th) in the dental bone defect and increased both bone and cementum formation in rats [184].

Jungbluth P et al., combined human iPSC-derived MSCs with calcium phosphate granules (CPG) in critical-size defects in the proximal tibias of minipigs. They demonstrated that iMSCs regenerated significantly better bone formation than the CPG scaffold alone and similar bone formation to that of an autologous BMSC [185]. Yu L et al., used retinoic acid to rapidly differentiate human iPSCs to the osteogenic lineage, and then combined them with a three-dimensionally printed Ti6Al4V (3DTi) scaffold for bone repair in a 5 mm mandibular bone defect that was generated in rats. Their results showed that iPSCs can form osteocytes in 10 days and enhance bone regeneration and the osseointegration of scaffolds [186]. Zhou ML et al., isolated bone marrow MSCs from patients with osteonecrosis of the femoral head (ONFH) or fracture tissues of the femur head, reprogramed them into iPSCs, and then derived MSCs from the iPSCs. They demonstrated that the proliferation of iPSC-MSCs was higher, and no tumorigenic ability was exhibited. When injected into the bone marrow cavity of rats with ONFH, they were as effective as the normal BMSCs in preventing bone loss and promoting bone repair in rats’ MPS-induced necrosis [187]. Zhou L et al., reprogramed human urine-derived cells into iPSCs and then derived MSCs from these iPSCs. Then, they combined the MSCs with hydroxyapatite-zirconia (HA/ZrO2) for bone repair in a rat skull defect model. The results showed that MSCs derived from iPSCs displayed the phenotypes and properties of normal BMMSCs, as well as the ability to proliferate and differentiate into osteogenic lineage, while promoting bone regeneration on rat skull defects in vivo [188]. Kato H et al., reprogramed mononuclear cells from human peripheral blood and then induced osteoblasts from these iPSCs, which revealed that these osteoblasts expressed osteoblast-specific markers. When they were transplanted into rat critical-size calvaria bone defect with collagen sponge scaffolds, the cell transplant group showed superior bone formation compared to those of the scaffold-only group [189].

### 12.2. IPSCS-MSC-Derived Exosome for Bone Tissue Engineering

Qi X et al., reported that human iPSCs-MSC-derived exosome promoted osteogenic differentiation of rat BMMSCs that were isolated from ovariectomized rats. IPSCs-MSC exosomes loaded on a β-TCP scaffold enhanced bone regeneration (higher BV/TV) and angiogenesis compared to scaffold-only in rat critical-size calvarial bone defects in ovariectomized rats [190].

In summary, iPSC-derived MSCs are new source of stem cells for promoting bone tissue engineering and are summarized in Supplemental Table S1.

## 13. Comparison of Bone-Regenerative Potential of Different Stem Cells

Given that stem cells can be isolated from almost every tissue, which cells are more effective remains a critical and unanswered question. Several studies have compared different sources of stem cells for bone regeneration.

### 13.1. BMMSCs Are Better Than ADSCs for Bone Tissue Engineering

Hayashi et al., compared the osteogenic differentiation of rat MSCs from bone marrow, periosteum, and adipose tissues in vitro and in vivo and found that MSCs from bone marrow and the periosteum were more osteogenic and formed significantly more bone in vivo than ADSCs [191]. Stockmann P et al., demonstrated that culture-expanded PSCs were as efficient as ADSCs and BMMSCs in terms of treatment of unicortical calvarial defects [192]. Another group found that, in critical-size sheep tibiae defects, ADSCs were not as efficient as BMMSCs for defect healing when seeded within a collagen scaffold, although they were much more efficient in bone healing when combined with platelet-rich plasma [193]. Xu L et al., compared the human BMMSCs and ADSCs isolated from bone marrow and adipose tissue obtained after total hip arthroplasty patients for epigenetic differences and in vivo bone regeneration. They demonstrated that BMMSCs regenerated more bone in a critical-size bone defect model in mice than ADSCs, likely due to their intrinsic epigenetic regulation of osteogenic and adipogenic genes [194]. Mohamed-Ahmed S et al., compared the osteogenesis of human ADSCs and BMMSCs from the same donors using poly(L-lactide-co-ε-caprolactone) scaffolds both in vitro and in vivo. They found that both ADSCs and BMMSCs demonstrated mineralization in vitro. However, BMMSCs showed higher ALP activity than ADSCs. In vivo, defects with BMMSC-seeded scaffolds had higher cellular activity than defects with ADSC-seeded scaffolds. Moreover, the bone formation in defects with BMMSC-seeded scaffolds was greater than it was in defects with ADSC-seeded scaffolds, especially at the early timepoints. These results suggest that although ADSCs have the potential to regenerate bone, the rate of bone regeneration with ADSCs may be slower than with BMMSCs [195].

### 13.2. ASDSCs Are More Efficient at Promoting Bone Repair Than BMMSCs and Similar to DPSCs

Mohammed EEA compared human AFDSCs and BMMSCs for their repair capacities in rat lumbar spine defects using a gel–foam scaffold. The results showed that human AFDSCs are more effective than the human BMMSCs for spinal fusion repair [196].

Maraldi T et al., compared DPSCs and AFDSCs for bone regeneration in critical-size calvarial bone defects using collagen as a scaffold. The authors found that both DPSCs and AFDSCs promoted bone regeneration by direct differentiation into osteoblasts while increasing blood vessel formation in the regenerated bone area using human mitochondria as a tracing marker [100].

### 13.3. DPSCs Exhibit Similar Bone Regeneration as BMMSCs

Nakajima K et al., compared stem cells from human exfoliated deciduous teeth (SHED) to that of human DPSCs and BMMSCs for bone regeneration using a polylactic-coglycolic acid barrier membrane as a scaffold in 4 mm calvaria defects of immunodeficient mice. Micro-CT results showed that the degree of bone regeneration with SHED in the bone defect was almost equivalent to that with human DPSCs and BMMSCs 12 weeks after transplantation. The ratio of new bone formation relative to the pre-created bone defect was not significantly different among groups with SHED, hDPSCs, and hBMMSCs. In addition, the histology demonstrated that SHED produced the greatest amount of osteoid and widely distributed collagen fibers compared to the human DPSC and BMMSC groups. Thus, SHED transplantation exerted a bone regeneration ability that was sufficient for the repair of bone defects [197]. Lee Y et al., compared BMMSCs’ and DPSCs’ cell morphology, cell proliferation, trilineage differentiation, mineral synthesis, and osteogenic gene expression in vitro and their bone regeneration in vivo using Bio-Oss^®^ as a scaffold. It was shown that the BMMSCs and DPSCs exhibited similar morphology, proliferative ability, surface marker profile, and trilineage differentiation potential in vitro. However, the BMMSCs exhibited a higher mineral deposition and expression levels of osteogenic marker genes, including ALP, RUNX2, and osteocalcin (OCN) [198]. In the in vivo studies, the new bone volume density in both cells groups was significantly greater than that in the empty control or Bio-Oss^®^-only group. Moreover, the new bone formation and Collagen I/osteoprotegerin protein expressions of the Bio-Oss^®^ BMMSCs or Bio-Oss^®^-DPSCs groups were higher than those of the Bio-Oss^®^-only group [198]. Finally, the Bio-Oss^®^+BMMSCs and Bio-Oss^®^+DPSCs groups had a similar bone mineral density, new bone formation, and osteogenesis-related protein expression [198]. Vater C et al., also compared DPSCs and BMMSCs for proliferation and bone regeneration in a critical-size calvarial bone defect. The authors found that DPSCs showed a 2-fold lower population doubling time and a 9-fold increase in proliferation when seeded onto mineralized collagen matrix (MCM) scaffolds compared to BMMSCs, but DPSCs showed a significantly lower osteogenic capability than BMMSCs. However, the pre-seeding of MCM scaffolds with DPSCs and BMMSCs did not enhance bone defect healing in vivo, as the healing of the critical-size bone defect in NMRI nude mice was comparable among all groups [199]. Another study compared the bone regeneration of the DPSCs and BMMSCs using MBCP and Bio-Oss^®^ scaffolds in a rabbit calvarial bone defect model. Despite the inferior bone-regenerative capacity of DPSCs and BMMSCs at early time points after bone injury compared to autologous bone grafting, at 8 weeks post-operatively, the efficiency of the BMMSCs combined with MBCP and Bio-Oss^®^ was comparable to that of the autogenous bone. DPSCs in combination with both scaffolds showed slightly inferior bone formation compared to autologous bone grafting [200].

### 13.4. ADSCs Are Better Than DPSCs for Bone Regeneration

Zhu Y et al., compared ADSCs and DPSCs for bone regeneration using bovine-derived xenografts with 10% porcine collagen as a scaffold. The study found that although DPSCs had higher proliferative abilities, ADSCs exhibited greater mineral depositions and higher osteogenic-related gene expression, indicating a better osteogenic differentiation potential of ADSCs [201]. After applying cryopreserved ADSCs and DPSCs in a critical-size calvarial defect model, both cryopreserved mesenchymal stem cells significantly improved the bone volume density and new bone area at 2, 4, and 8 weeks. Furthermore, the combined treatment with ADSCs and xenografts was more efficient in enhancing bone repair compared to the combined treatment with DPSCs at all time points [201]. The authors further evaluated the sequential early bone healing process both histologically and radiographically, confirming a high level of agreement between these two methods, which supports the conclusion [201].

### 13.5. MDSCs Are Similar to BMMSCs for Bone Regeneration

Gao X et al., compared the bone regeneration of Lenti-BMP2-transduced human BMMSCs and MDSCs in a critical-size mouse calvarial bone defect model using fibrin sealant as a scaffold. The authors found both Lenti-BMP2-transduced BMMSCs and MDSCs regenerated functional bone in 6 weeks, with near complete defect healing. No significant differences were found in terms of the new bone volume and defect healing percentage between Lenti-BMP2-transduced human BMMSCs and MDSCs [58]. However, non-transduced human BMMSCs and MDSCs all formed negligible amounts of new bone, which indicated that BMP2 signaling is required [58]. Lough D et al., compared MDSCs with ADSCs and BMMSCs isolated from the same mice for bone regeneration. The authors found that while all populations exhibited mesenchymal stem cell multilineage capacity, ADSC- and BMMSC-enriched constructs were capable of forming small bone aggregates. In contrast, MDSCs self-assembled a form of organized cortico-cancellous bone structures within two- and three-dimensional in vitro systems. MDSCs also augmented defect healing, angiogenesis, and diploic space formation in a cranial defect mice model in vivo [202].

### 13.6. PSCs Are More Efficient Than BMMSCs for Bone Regeneration

Agata H et al., compared the bone regeneration capacities of BMMSCs and PSCs and found that PSCs were capable of osteogenic differentiation in vitro, although less efficiently than BMMSCs; however, when PSCs were pretreated with FGF2 and BMP2, they induced greater bone formation in vivo when compared to the BMMSCs [203]. González-Gil AB et al., compared the therapeutic potential of PSCs and BMMSCs in combination with biomaterials in a bone non-union model. PSCs, BMMSCs, and bone graft were isolated from green fluorescent protein (GFP)-transgenic rats. Animals were divided into six groups. It was found that in the live bone allograft (LBA) group, all the animals showed bone bridging (n = 6), whereas in the CSBMP2 group, four out of six animals demonstrated healing. In the PCL and PCLPSC groups, a reduced number of animals showed radiological healing, whereas no healing was detected in the PCL-BMMSC group. Micro-CT results showed significant new bone formation in the LBA, CSBMP2, and PCL-PSC groups when compared with the CTL group. Finally, tracking of cellular implants demonstrated significantly higher survival of the PSCs when compared with BMMSCs [204].

In summary, BMMSCs are still the most commonly used postnatal stem cells. BMMSCs are more efficient than ADSCs for bone regeneration and are equivalent to MDSCs, but slightly better than DPSCs. AFDSCs and PSCs are more effective than BMMSCs and comparable to DPSCs for bone regeneration.

## 14. Advantages and Disadvantages of Different Stem Cells for Potential Clinical Applications

**Figure 1:**
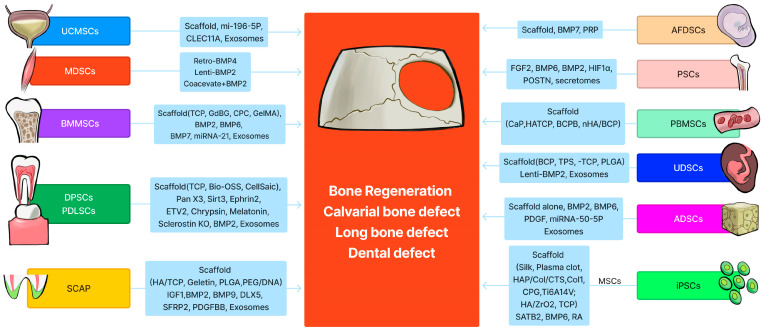
Schematic summary of 12 different stem cells for bone tissue engineering. The graph was created by Xiang Xiao using Figma (https://www.figma.com, accessed on 11 February 2024).

In summary, many sources of stem cells are available for bone tissue engineering. The source of the stem cells to be utilized in clinical practice will depend on the patient’s needs and the availability of donor tissues. BMMSCs are the most commonly used and overall are most effective in terms of promoting bone regeneration. PSCs are also as effective as BMMSCs, but their isolation procedures are invasive [192]. MDSCs are often available during treatment of orthopedic trauma, can be isolated with easy muscle access, and regenerate functional bone efficiently, although they require BMP4 or BMP2 stimulation [58]. ADSCs are readily available at the point of care despite being less effective than BMMSCs. DPSCs are also attractive due to their near-similar efficacy compared with BMMSCs for bone regeneration [201]. They represent a great cell source for dental bone loss or craniofacial bone reconstruction. UC-MSCs are also promising because their harvest is not invasive [139]. They can be used for both mother and infant (children) treatment and allow for stem cell banking for later use. Most of the recent studies on PBMSCs for bone regeneration are also encouraging due to their effectiveness and relative ease of availability for isolation. Recent investigations on UDSCs are especially encouraging in that they are effective, readily available at the point of care, non-invasive, inexpensive to isolate, and suitable for stem cell banking. Applications of different stem cells for different bone defect repairs are summarized in Figure 1 and Supplemental Table S2.

## 15. Prospective Applications of Stem Cells in Bone Tissue Engineering for Human Bone Tissue Repair

Despite extensive preclinical studies using many different stem cells from different tissue resources, the clinical applications of stem cells are still limited due to most of the stem cells needing culture and expansion. Therefore, the development of new methods or devices allowing for the point-of-care isolation of stem cells for bone defect repair or non-union fracture repair is critical. For example, Zhang Y et al., reported a point-of-care device for isolating and processing BMMSCs, forming a composite with a scaffold in 5 min, which achieved clinically satisfactory bone repair for 42 patients [205]. Furthermore, stem cell banking of different stem cells for future application is also an important strategy. UC-MSCs, PBMSCs, UDSCs, DPSCs, MDSCs, ADSCs, and BMMSCs are all excellent stem cell resources that are also suitable for stem cell banking.

The choice of scaffold to deliver cells is also important. Fibrin sealant scaffolds are FDA-approved (such as Tisseel Fibrin Sealant) and, when used for delivery of cells or growth factors, are easily absorbable, with the newly formed bone being similar to native bone, with normal blood vessel and bone marrow anatomy [58,60,206]. BCP or TCP or hydroxyapatite scaffolds are not easily absorbable and often remain in the newly regenerated bone; these residues do not integrate with the host bone and likely offer inferior bone biomechanical properties. Bioactive growth factor peptide-conjugated scaffolds may be more suitable to deliver with stem cells, with better safety than gene delivery approaches [33]. Ideally, stem cell-based strategies in bone tissue engineering will need the stem cells from both the donor and the host to differentiate into osteoblasts, secrete collagen I and other organic bone matrix components, and then mineralize to form fully functional bone. Scaffolds that are used for each category of stem cells are summarized in Supplemental Table S1. The orchestration of osteogenesis with angiogenesis is also important. The combination of stem cells, their secretome, bone growth factors, and bio-engineered scaffolds will be highly effective. Finally, exosomes or extracellular vesicles derived from stem cells offer a cell-free strategy of delivering osteogenic cargos to enhance bone formation. This approach is very promising due to its nature of not eliciting an immune response when used allogenically.

## Data Availability

Data sharing is not applicable.

## Supplementary Materials

The following supporting information can be downloaded at https://www.mdpi.com/article/10.3390/life14030287/s1: Table S1. Summarized bone formation factors and scaffolds used for different stem cell-mediated bone formations; Table S2. Summarized stem cells for different bone defect repairs with references.
