# Supplementary material for: Stem Cells and Bone Tissue Engineering
Source: Life (Basel). 2024 Feb 21;14(3):287. doi: 10.3390/life14030287 (PMC10971350; doi:10.3390/life14030287)
Supplement: Supplementary file 1 [file life-14-00287-s001.zip › life-2834690-supplementary.pdf]

Supplemental Table S1: Summarized bone formation factors and scaffold used for different stem cell mediated bone formation.

Stem cells Sources	Growth factors or functional small molecules	Scaffold	Bone regeneration effects	Cited references
BMMSCs	miRNA21	TCP	Enhanced bone repair	[12, 13, 15]
		Gadomium-bioglass with chitosan microscphere	Enhanced defect repair	[14]
		Calcium deficient hydroxyapatite	Regenerated bone better than TCP	[16]
	PRP	Calcium phosphate cement (CPC)	Regenerated bone better than CPC	[17]
		Graphene oxide methacrylated gelatin (GO-GelMA) or silica-coated graphene oxide methacrylated gelatin (SiGO/GelMA)	Regenerated bone better than GelMA	[18]
		HBMMSCs derived extracellular matrix	Enhanced bone formation	[19, 25]
		GO modified Silk fibroin	Promoted osteogenic differentiation	[20]
		Photo crosslinked GelMA	Increased bone formation	[22]
		Rotary jet spinning (RJS) PCL carbon nanotube	Significant increased bone formation	[23]
		Hetero-nanolayer with black phosphus and GO	Improved defect healing	[24]
		Fibrin-bioink-PCL fiber	Enhance bone healing better than Collagen	[27]
		Hydroxyapatite mineralized Antheraea pernyi fibroin	Increased bone matrix proteins in vivo	[28]
		Air-plasma treated silk fibroin	Increased bone matrix in ectopic model	[29]

	TGF-β1+BMP2	MSCs tube	More robust bone formation than MSCs sheet	[30]
	BMP6	Nano-hydroxyapatite (nHA)/gelatin (Gel)/gelatin microsphere (GMS)	Significantly accelerated bone regeneration	[31]
	BMP2	Hydroxyapatite viscoelastic gel	Enhanced bone formation	[32]
	BMP2 peptide	BMP2 peptide fused to Bombyx mori silk fibroin	Stimulated bone formation in subcutaneous model	[33]
	Exosome	MeGC hydrogel ,3D-printed titanium alloy scaffold, mesoporous bioactive glass (MBG), fibronectin (RGD)	Enhanced calvarial and long bone defect	[34-37]
MDSCs	Adeno-BMP2,<br><br>retro-BMP2,BMP4<br><br>Lenti-BMP2	Collagen sponge, Fibrin sealant	Completely healed defect in 4-6 weeks	[45, 48-55, 57-62]
ADSCs		PLGA scaffold	Enhanced Complete healing	[66]
		Fibrin sealant	Near complete healing	[67]
		Hydroxyapatite (HA)-poly(lactic-co-glycolic acid	Enhanced bone defect healing by fresh HADSCs not frozen HMDSCs.	[68]
		PCL/β-tricalcium phosphate (β-TCP) scaffolds	Enhanced radial defect healing	[69]
		Hydroxyapatite/poly(lactide-co-glycolide)	Not significant improved bone healing using rat ADSCs	[70]
		Decellularized bone scaffold	7/8 femur bone defect bridging	[71]

		Heterogenous deproteinized bone matrix	Completely regenerated radial defect after pre-osteogenic differentiation in vitro	[72]
		Polylysine (PLL)-modified coralline hydroxyapatite (CHA)	Completely healed the radial defect with lamellar bone	[74]
		Tricalcium phosphates (TCP) and a PLGA scaffold	Regenerated bone bone in mandible defect than no cell control.	[75]
	Adeno-BMP2	Collagen ceramic carrier	Completely healed femur defect compared to no healing with cells only.	[76]
	Baculovirus-BMP2 and CRISPRi-Noggin	Spongostan gelatin sponge	Enhanced bone defect healing in calvarial bone defect	[77]
	BMP2	Apatite-coated porous poly(l-lactide-co-dl-lactide)	Enhanced femoral bone defect healing, but not better than BMP2 alone.	[78]
	BMP7	PCL/β-TCP/DBM	Significantly enhanced bone regeneration in canine femur bone defect	[79]
	BMP6	N/A	Formed bone ossicle after in vitro chondrogenic differentiation	[80]
	PDGF	Bio-mineral coated fibers	Enhanced bone healing	[81]

			in calvarial bone defect	
	BMP2	N/A	Enhanced bone regeneration in osteonecrosis femur head	[82]
	Phenamil+BMP2	Poly(lactic-co-glycolic acid) and apatite layer	Enhanced calvarial defect healing	[83]
	LLP2A-Alendronate (LLP2A-Ale)	N/A	Enhanced femur facture healing by attract ADSCs to fracture site	[84]
	DKK1 antibody	N/A	Promoted femur defect healing by increase ADSCs survival and engraftment	[85]
	Hedgehog modifier(recombinant N-terminal Sonic hedgehog, smoothened agonist, and cyclopamine).	Apatite-coated poly(lactic-co-glycolic acid) (PLGA) scaffolds	Enhanced calvarial bone defect healing	[86]
	Human Exosome	Polydopamine-coating poly (lactic-co-glycolic acid) (PLGA/pDA)	Enhanced calvarial bone defect healing	[87]
	Human miR-375 enriched exosome	Thiol‐modified hyaluronan, hydroxyapatite and thiol‐modified heparin	Promoted calvarial bone defect healing	[88]
	Human Exosome	PLGA/Exo-Mg-GA metal-organic framework (MOF)	Promoted new bone formation	[89]
	Rat exosome	DMPE-PEG-CREKA	Enhanced bone repair	[90]
	miR-450b,	N/A	Enhanced ectopic bone formation	[91]
	miR-150-5p	Hydroxyapatite/tricalcium phosphate (HA/TCP)	Enhanced bone formation by inhibiting	[92]

			miRNA0150-5P	
DPSCs/PDLSCs		N/A	Increased Lamella bone	[97]
		Hydroxyapatite/TCP scaffolds	Increased bone formation in vivo	[98]
		Collagen gel constructs	Improved calvarial bone defect healing	[100]
		Bio-Oss® scaffold	Repaired bone defect of rabbit alveolar toothless jaw	[101]
	BMP2	Tyrosine-derived polycarbonate polymer scaffolds [E1001(1k)] containing beta-tricalcium phosphate	Enhanced mandibular ramus critical bone defect healing but less than BMP2	[102]
	Combined with HUVEC	Tyrosine-derived polycarbonate polymer scaffolds [E1001(1k)] containing beta-tricalcium phosphate	Enhanced jaw bone defect healing than hDPSCs alone	[103]
		β-tricalcium phosphate	Enhanced human periodontal defect healing	[104]
		DPSC cells sheet	Improved miniature pig periodontitis with bone defects	[105]
		DPSC-CellSaic	Improved rat congenital cleft fracture bone formation	[106]
		Ceramic nanocomposites of hydroxyapatite/titania /calcium silicate	Improved rabbit tibia defect healing	[107]
		Collagen matrix	No benefit for human post-extraction	[108]

			sockets of impacted mandibular third molars	
	Lenti-Pannexin3	β-TCP scaffold	Increased bone formation in critical size calvarial bone defects	[109]
	Adeno-SIRT1	N/A	Enhanced distraction osteogenesis	[110]
	Lenti-ephrinB2	PuraMatrix Peptide Hydrogel	Enhanced canine alveolar bone formation and quality	[111]
	ETV2	β-TCP scaffold	Enhanced rat calvarial bone defect healing and mice ectopic bone formation	[112]
	helioxanthin derivative, 4-(4-methoxyphenyl) pyri-do[40,30:4,5]thieno[2,3-b]pyridine-2-carboxamide (TH)	Cell sheet	Enhanced tibia fracture repair	[113]
	chrysin	β-TCP scaffold	Enhanced ectopic bone formation and calvarial bone defect healing	[114]
	melatonin	MBCP scaffold	Reparied Better than scaffold control but not DPSC only.	[115]
	SOST ab or Knockout SOST in cells	Collagen hydrogel	Improved bone rdegeneration in WT mice and SOSTKO mice	[116]
	Human PDLSCs exosome	β-TCP scaffold	Accelerated bone	[117]

			formation in alveolar bone defects	
	Human Extracellullar vesicles	Matrigel	Enhanced alveolar bone defect healing	[118]
PSCs	FGF2	Calcium phosphate-collagen scaffold	Completely healed femoral defect	[120]
	BMP6	Calcium phosphate scaffold with intermediate release of Ca2+	Robust bone formation in ectopic bone model	[121]
	BMP2 or BMP6	Ceramic dicalciumphosphate scaffold (CopiOs®)	Promoted sheep 4.5cm biological exhausted tibia defect healing	[122]
	Periostin	N/A	Rescued HIFa KO PSC bone regeneration	[123]
		N/A	Promoted bone formation in ectopic bone formation model.	[124]
	Secretome	Bioceramic xenograft scaffold	Promoted rabbit calvarial bone defect healing	[126]
AFDSCs	BMP7	Nanofibrous scaffolds	Enhanced bone formation in subcutaneous ectopic bone formation	[127]
		Collagen scaffold	Enhanced calvarial defect healing	[100]
		30% Nano-hydroxyapatite chitosan scaffold	Completely healed rabbit defect healing in 4 weeks	[129]

		Human AFDSCs cell sheet	Enhanced calvarial bone healing	[130]
	PRP	N/A	Repaired alveolar bone defect	[131]
	PRP	Random polycaprolactone (PCL) fibrous scaffolds	Enhanced calvarial defect	[132]
PBMSCs		Porous calcium phosphate resorbable scaffold	Enhanced Ulna defect repair	[133]
		HA/TCP	Enhanced root canal defect healing	[134]
	Combine with peripheral epithelia cells	Biphasic calcium phosphate bioceramic (BCPB) scaffold	Enhanced rabbit long bone defect	[135]
	Combine with peripheral epithelial cells	3D-printed biphasic calcium phosphate (BCP) scaffold with highly bioactive nano hy-droxyapatite (nHA) coating (nHA/BCP)	Enhanced rabbit femur long bone defect	[136]
		Hydroxyapatite-poly(lactic-coglycolic acid) (HA-PLGA) scaffolds	Induced bone healing in calvarial bone defect	[137]
		Porous and resorbable β-tricalcium phosphate (β-TCP) scaffolds	Regenerated bone in ectopic bone formation model.	[138]
UC-MSCs		Biomimetic artificial bone scaffold	Formed bone in ectopic bone formation model	[139]
	pEGFP-OSX plasmid	PLGA scaffold	Promoted bone formation in subcutaneous ectopic bone formation model	[140]
		Intra-venous infusion	Decreased necrotic volume of ONFH of human	[141]
		Bio-Oss® scaffold	Significantly enhanced	[142]

			calvarial bone defect healing in nude rat	
	miR-196a-5p	N/A	Promoted calvarial bone defect healing	[143]
	Human Exosome	Injectable hydroxyapatite (HAP)-embedded in situ cross-linked hyaluronic ac-id-alginate (HA-ALG) hydrogel	Significantly enhanced bone regeneration in rat calvarial bone defect	[144]
	Human Exosome	Systemic injection	Prevented bone loss, maintain ed bone mass	[145]
	Human exosome	Chitosan/hydroxyapatite (CS/HA) scaffold	Regenerated significantly more bone in rat calvarial bone defect	[147]
UDSCs		β-TCP	Healed rat femur segmental defect	[148]
	Lenti-BMP2	β-TCP	Enhanced ectopic bone formation in rat muscle pocket model	[149]
		Calcium silicate (CS) particles incorporated into poly (lactic-co-glycolic acid) (PLGA) composite scaffolds	Enhanced ectopic bone formation in muscle pocket	[150]
		Surface mineralized biphasic calcium phosphate ceramics (BCPs)	Enhanced rabbit ulna segmental bone defect	[151]
		Graphene oxide-modified silk fibroin/nanohydroxyapatite scaffold	Completely healed rat calvarial bone defect healing	[152]
		Biphasic calcium phosphate (BCP) bioceramic ornamented with chitosan sponges (CS) (CS/BCP) hybrid scaffold	Promoted ulna segmental bone defect healing	[153]

	BMP2	Chitosan microspheres/type I collagen hydrogel (BMP2-CSM/Col I hydrogel) l	Enhanced bone formation in rat calvarial bone defect	[154]
		3D-printed poly(ε-caprolactone) (PCL) scaffold	Enhanced calvarial bone defect healing in rabbit	[155]
		3D-printed polylactic acid and hydroxyapatite (PLA/HA) composite scaffold	Enhanced rat calvarial bone defect healing	[156]
	Exosome		Decreased osteolytis and increased bone formation	[104]
	Exosome	Gelatin methacrylate (GelMA) and hyaluronic acid methacrylate (HA-MA)/nano-hydroxyapatite (nHAP) hydrogels	Promoted rat calvarial defect healing	[158]
SCAPs	Mini-Pig SCAP	HA/TCP	Formed bio-roots to support porcelain crown	[159]
	Human SCAP+IGF1	Absorbable gelatin sponges	Formed bone in renal ectopic bone formation model when treated with IGF-1 or form dentin when not treated with IGF-1	[161]
	Mouse SCAP-Adeno-BMP9	N/A	Formed bone and cartilage in dorsal flank ectopic bone formation model	[162]
	Mouse SCAP-AdenoBMP9 and Wnt3A	N/A	Formed significant more trabecular bone in dorsal flank	[163]

	Human SCAP-BMP2	PLLA or PLGA Nano Fiber-Microsphere	Formed bone in mouse dorsal flank	[164]
	Canine delivered with peripheral blood	N/A	Formed dentine tubule like structure in dog periapical periodontitis model	[165]
	Human SCAP	N/A	Enhanced periodontal tissue regeneration in mini-pig periodontitis Model	[166]
	Human SCAP-DLX5	N/A	Enhanced bone formation in mice dorsal flank ectopic bone formation model	[167]
	Human SCAP-SDF1α and BMP2	N/A	Enhanced bone formation in mice dorsal flank ectopic bone formation model	[168]
	Human SCAP-SFRP2	N/A	Enhanced bone formation in-mini-pig Periodontitis model	[169]
	Human SCAP-PDGFBB	Thermosensitive hydrogel	Enhanced calvarial bone defect in 5mm defects of SD rats	[170]
	Human SCAP exosomes+human BMMSCs	Gelatin sponge	Enhanced bone formation in mice dorsal flank model	[171]

	Human SCAP exosome	Bioresponsive polyethylene glycol (PEG)/DNA hybrid hydrogel triggered by MMP9	Enhanced mandible bone defect in SD rates	[172]
	Human SCAP exosome stimulated with lower Intensity pulsed ultrasound	N/A	Improved Bone healing in mouse periodontitis model	[173]
iPSCs	SATB2 over expression mouse iPSCs	Silk scaffold	Improved calvarial bone defect healing	[179]
	Non-human primate iPSCs derived mesoderm cells	Plasma clot	Formed new bone without teratoma formation	[180]
	Mouse iPSCs derived MSCs	Biomimetic nanofibers of hydroxyapatite/collagen/chitosan (HAp/Col/CTS) scaffold	Increased bone regeneration 2-fold	[181]
	Human iPSCs derived early and later MSCs/plasmid-BMP6	Collagen type I biodegradable scaffolds	Repaired rabbit radial defect as efficient as bone marrow MSCs	[182]
	Human iPSCs derived mesoderm cells grown cartilage pellets	N/A	Near complete healing of 5mm calvarial defect in rats	[183]
	Rat iPSCs culture in BMP6 osteogenic medium	Chitosan/gelatin/glycerol phosphate hydrogel	Increased bone and cementum formation in maxillary-molar defects	[184]
	Human iPSCs-derived MSCs	Calcium phosphate granules (CPG)	Regenerated significantly better bone than scaffold and similar as autologous BMC in mini-pig critical size tibia defect	[185]
	Human iPSCs differentiated in retinoic acid	Three-dimensionally printed Ti6Al4V (3DTi) scaffold	Formed osteocytes in 10 days in rat	[186]

			mandibular bone defect.	
	Human ONFH derived iPSCs derived MSCs	N/A	Prevented bone loss and repair of rat ONFH	[187]
	Human urine derived cells generated iPSCs derived MSCs	Hydroxyapatite-zirconia (HA/ZrO2)	Promoted bone regeneration in rat skull defect	[188]
	Human peripheral blood derived iPSCs derived MSCs	Collagen sponge scaffolds	Superior bone regeneration than scaffold group in rat critical size calvarial bone defect model	[189]
	Human iPSCs derived MSCs derived exosome	β-TCP scaffold	Promoted bone regeneration and angiogenesis in critical size calvarial bone defect in ovariectomized rats	[190]

Note: References number match the reference number in the main text.

Supplemental Table S2. Summarized stem cells for different bone defect repair with references.

Stem cells category	Species of stem cells	Calvarial defect	Long bone defect	Maxillary/mandible	Other models
BMMSCs	Human	Enhanced bone defect healing [14, 16, 32]	Enhanced condyle defect [12], or femur defect healing [27, 30]		Increased bone matrix Ectopic bone model [28, 29, 33]
	Mouse		Ehhanced Tibia defect [26]		
	Rat	Enhanced defect healing [13, 23, 24, 31]	Robust bone formation [22]		
	Mini-pig		Enhanced defect healing [17]		

	Canine MSCs	Enhanced defect healing [13]	Not as efficient as fresh MNMSCs [15]	Enhanced alveolar bone regeneration [13]	
BMMSCs exosome	Human	Robust bone regeneration [34, 37]	Promoted bone regeneration [35]		
	Rat	Robust bone regeneration [36]			
MDSCs	Human	Significantly enhanced bone regeneration [55, 57-59]			Increased bone formation in Ectopic bone model [61, 62]
	Mouse	Complete bone defect healing in 4-6 weeks [45, 48-54, 60]			
ADSCs	Human	Near complete healing [67, 68, 81]	7/8 femur bone defect bridging [71, 76, 78, 85]		Generated bone ossicle Ectopic bone model [80]
	Mouse	Significantly enhanced bone healing [66, 83]	Enhanced femur fracture healing [84]		
	rat	Significantly enhanced bone healing [77]	Complete bridging radial defect [72]		
	Canine		Significantly enhanced radial bone defect healing [69, 79]		
	Rabbit		Radial bone defect complete healed with lamellar bone[74]		Promoted osteonecrosis femur head (ONFH) regeneration [82]
	Mini-pig			Regenerated more bone than no cell group [75]	
ADSCs exosome	Human	Enhanced defect healing			

		but not healed [87-89]			
	Rat small Extracellualar vesicle		Enhanced femur defect healing[90]		
ADSCs-miRNAs	Human, miR-450b overexpression, miR-150-5p inhibition				Enhanced ectopic bone formation[91, 92]
DPSCs	Human	Enhanced calvarial bone defect healing [100, 109, 112, 114]	Improved rabbit tibia defect [107] Promoted Tibia fracture healing [113]	mandibular ramus critical bone defect [102-104], miniature pig periodontitis with bone defects[105]	Enhanced ectopic bone formation [97, 114]; Enhanced distraction osteogenesis[110]
	Rat			Promoted rat mandibular congenital defects [106]	Enhanced bone formation [98]
	Rabbit	Enhanced calvarial bone defect healing than scaffold [115]		Enhanced alveolar bone formation [101]	
	Canine			Enhanced alveolar bone formation [111]	
	Mouse	Enhanced calvarial bone defect healing [116]			
	Human PDLSCs exosome or extracellular vesicles			Enhanced alveolar bone formation [117, 118]	
PSCs	Human				Robust bone formation in ectopic formation model [121, 124]
	Mouse		Complete healing of femoral defect [120]		
	Sheep		Enhanced healing in 3cm defect		

			4.5cm tibia defect healing when combined with BMP2 [122]		
	Rat		Increased implant integration in femur [125]	Increased implant integration in mandible defect [125]	
	Rabbit	Enhanced calvarial bone defect [126]			
AFDSCs	Human	Enhanced calvarial bone defect healing [100, 130]	Completely healed rabbit tibia defect in 4 weeks [129]		Enhanced bone formation in subcutaneous ectopic bone [127]
	Rat	Enhanced calvarial bone defect repair [132]		Repaired alveolar bone defect [131]	
PBMSCs	Human				Regenerated bone in ectopic bone formation model[138]
	Rabbit		Enhanced rabbit Ulna defect [133, 135, 136]	Repaired mouse mouse root canal [134]	
	Mouse	Induced bone formation in mouse calvarial bone defect[137]			
UC-MSCs	Human	Significantly promoted calvarial bone defect healing [142, 143]			Formed bone in ectopic bone formation model [139, 140], Decreased necrosis volume of ONFH in human [141]
	Human Exosome	Enhanced rat calvarial bone defect healing [144, 147]			Prevented bone loss increased bone strength [145]
UDSCs	Human	Completely healed calvarial bone	Healing of rat femur segmental		Enhanced ectopic bone formation in

		defect in 12 weeks [152] or enhanced defect healing [154-156]	bone defect[148, 151]		muscle pocket [149, 150]
	Human exosome	Promoted rat calvarial bone defect healing [158]			Decreased osteolytis and increased bone formation [157]
SCAP	Mini-Pig			Promoted Bio-root formation to support porcelain crown [159]	
	Human	Enhanced calvarial bone defect healing in SD rats [170]		Promoted periodontal tissue regeneration in a mini-pig-periodontitis model [166, 169]	Formed ectopic bone in renal capsule when treated with IGF-1, form dentin when use SCAP only [161, 164, 167, 168]
	Mouse				Formed bone in dorsal ectopic bone formation model [162, 163]
	canine			Formed dentine tubule like structure in dog periapical periodontitis model [165]	
	Human SCAP exosome +human BMMSCs	Enhanced mandible defect healing in rat[172]			Increased ectopic bone formation in mice dorsal flank model [171]
	Human SCAP exosome stimulated with lower Intensity pulsed ultrasound			Improved bone healing in mice periodontitis [172]	
iPSCs	Mouse iPSCs transduced with SATB2	Enhanced calvarial bone defect healing [179]			
	Primate iPSCs				Enhanced bone formation in mice dorsal subcutaneous

					ectopic bone formation model [180]
	Mouse iPSCs derived MSCs	Enhanced calvarial bone defect healing [181]			
	Human iPSCs derived iPSCs, BMP6	Enhanced rat calvarial bone defect using cartilage pellets [183]	Complete healing of radial defect in rabbit [182]		
	Rat iPSCs cultured in BMP6 osteogenic medium			Promoted bone and cementum formation in maxillary-molar defects [184]	
	Human iPSCs derived MSCs		Regenerated significantly better bone than scaffold and similar as autologous BMC in mini-pig critical size tibia defect [185]	Promoted rat mandible bone defect [186]	
	Human iPSCs derived from ONFH derived MSCs				Prevented bone loss and bone repair in ONFH rat model [187]
	Human urine derived cells generated iPSCs derived MSCs	Promoted bone regeneration in rat skull defect [188]			
	Human peripheral blood mononuclear cells derived iPSCs derived osteoblasts	Superior bone formation in rat critical size calvarial bone defect [189]			
	Human iPSCs derived exosome	Enhanced bone regeneration and angiogenesis			

		is ovariectomized rats calvarial bone defect model [190]			

Note: Reference numbers match reference number in the main text.
